# Dynamic clonal equilibrium and predetermined cancer risk in Barrett's oesophagus

**DOI:** 10.1038/ncomms12158

**Published:** 2016-08-19

**Authors:** Pierre Martinez, Margriet R. Timmer, Chiu T. Lau, Silvia Calpe, Maria del Carmen Sancho-Serra, Danielle Straub, Ann-Marie Baker, Sybren L. Meijer, Fiebo J. W. ten Kate, Rosalie C. Mallant-Hent, Anton H. J. Naber, Arnoud H. A. M. van Oijen, Lubbertus C. Baak, Pieter Scholten, Clarisse J. M. Böhmer, Paul Fockens, Jacques J. G. H. M. Bergman, Carlo C. Maley, Trevor A. Graham, Kausilia K Krishnadath

**Affiliations:** 1 Evolution and Cancer Laboratory, Centre for Tumour Biology, Barts Cancer Institute, Queen Mary University of London, EC1M 6BQ London, UK; 2 Department of Gastroenterology and Hepatology, Academic Medical Center—University of Amsterdam, 1100 DD Amsterdam, The Netherlands; 3 Center for Experimental and Molecular Medicine, Academic Medical Center—University of Amsterdam, 1100 DD Amsterdam, The Netherlands; 4 Department of Pathology, Academic Medical Center—University of Amsterdam, 1100 DD Amsterdam, The Netherlands; 5 Department of Gastroenterology and Hepatology, Flevoziekenhuis, 1300 EG Almere, The Netherlands; 6 Gastroenterological Association, 1006 AE Amsterdam, The Netherlands; 7 Department of Gastroenterology and Hepatology, Tergooiziekenhuizen, 1201 DA Hilversum, The Netherlands; 8 Department of Gastroenterology and Hepatology, Medisch Centrum, 1800 AM Alkmaar, The Netherlands; 9 Department of Gastroenterology and Hepatology, Onze Lieve Vrouwe Gasthuis, 1091 AC Amsterdam, The Netherlands; 10 Department of Gastroenterology and Hepatology, Sint Lucas Andreas Ziekenhuis, 1006 AE Amsterdam, The Netherlands; 11 Department of Gastroenterology and Hepatology, Spaarne Ziekenhuis, 2134 TM Hoofddorp, The Netherlands; 12 Biodesign Institute, School of Life Sciences, Arizona State University, Tempe, Arizona 85281, USA

## Abstract

Surveillance of Barrett's oesophagus allows us to study the evolutionary dynamics of a human neoplasm over time. Here we use multicolour fluorescence *in situ* hybridization on brush cytology specimens, from two time points with a median interval of 37 months in 195 non-dysplastic Barrett's patients, and a third time point in a subset of 90 patients at a median interval of 36 months, to study clonal evolution at single-cell resolution. Baseline genetic diversity predicts progression and remains in a stable dynamic equilibrium over time. Clonal expansions are rare, being detected once every 36.8 patient years, and growing at an average rate of 1.58 cm^2^ (95% CI: 0.09–4.06) per year, often involving the *p16* locus. This suggests a lack of strong clonal selection in Barrett's and that the malignant potential of ‘benign' Barrett's lesions is predetermined, with important implications for surveillance programs.

## Abstract (web summary)

Barrett's oesophagus is thought to be a precursor lesion for oesophageal cancer, and predicting the benign lesions that progress to cancer is clinically important. Here, the authors use FISH to study the clonal evolution of Barrett's oesophagus and show that genetic diversity and somatic mutations are present early in the benign disease.

## Graphical abstract

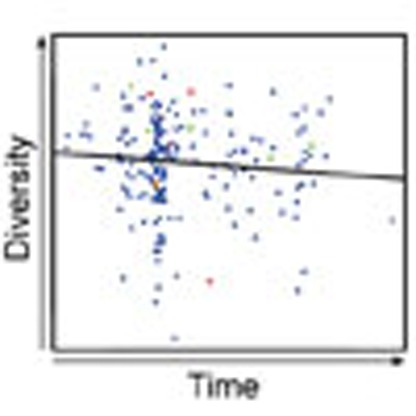

Carcinogenesis is fundamentally an evolutionary process, whereby cells that have acquired advantageous somatic mutations clonally expand via a process of Darwinian natural selection^1^,^2^. Initial models of this process, based on the organismal evolution and evidence that multiple mutations were required to generate a cancer, assumed progression was characterized by a series of clonal expansions, each sweeping to fixation within the tumour. Barrett's oesophagus (BE) provides an ideal condition to study the dynamics of somatic evolution over both space and time in humans *in vivo*^3^. However, despite extensive genomic analysis of BE and its associated esophageal adenocarcinoma (EAC)^4^,^5^,^6^, the dynamics of clonal evolution, including changes in the clonal diversity, as well as the frequency and rate of clonal expansions, have remained largely uncharacterized, as is the case for all neoplasms. The evolutionary dynamics of Barrett's clones are important both for understanding the fundamental process of neoplastic progression and for the clinical management of the disease.

BE is an acquired metaplastic epithelial change in the lower oesophagus, thought to be an adaptive response to chronic gastro-esophageal reflux^3^,^7^. Patients with BE have an increased risk of developing EAC^8^. However, the absolute risk of progression in particular for Barrett's patients without dysplastic changes is only 0.12–0.6% per year^8^,^9^ and most of these patients will never progress to EAC in their lifetime. Following current guidelines, BE patients are enrolled in endoscopic surveillance programs to detect dysplasia and early cancers that can be effectively cured by minimally invasive interventions^10^,^11^,^12^. Due to a lack of tools for robust risk stratification, and the costs involved, the clinical management and surveillance strategy for the large group of non-dysplastic Barrett's (NDBE) patients is debated. Measurements of the evolutionary process that drives progression may provide universal biomarkers for robust risk stratification. These evolutionary biomarkers could be universal in their applicability to virtually all types of neoplasms^2^,^13^,^14^,^15^.

Here we analyse multicolour fluorescence *in situ* hybridization (FISH) data to assess the genetic diversity at single-cell resolution in NDBE patients. We confirm that genetic diversity correlates with the risk of progression to cancer. Moreover, we show that the level of genetic diversity is invariant over time, suggesting an absence of strong selection in the evolution in NDBE and consequently that progression risk is predetermined by the invariant baseline level of diversity. Finally, we provide estimates of the *in vivo* rate of clonal expansion and contraction of mutant clones in a human metaplastic lesion.

## Results

### Multicolour FISH reveals genetic diversity on a single-cell basis

We performed DNA FISH analysis on endoscopic brush cytology specimens collected from 320 Barrett's patients who all had effective acid suppressive therapy and no morphological evidence of dysplasia at baseline. Patients were followed for a median of 43 months (range 11–130 months) during which 20 patients (6.3%) progressed; 8 patients developed high-grade dysplasia (HGD) and 12 developed EAC after a median duration of 34 months (Table 1). For 195 of these patients we also analysed material from a second endoscopy, which included 14 progressors (HGD, *n*=5 and EAC, *n*=9), and there was a median interval of 37 months between the first and second brushes (interquartile range: 34–52). A minimum of 50 cells (Supplementary Fig. 1) per sample were scored for abnormalities by FISH at seven markers including CEP7, CEP17, *p53, p16, Her-2/neu*, *20q* and *MYC*, organized into two probe sets (Methods). The adequacy of counting this number of cells to measure clone-size abundance and diversity was confirmed by bootstrap analysis (Supplementary Fig. 2).

**Figure 1:**
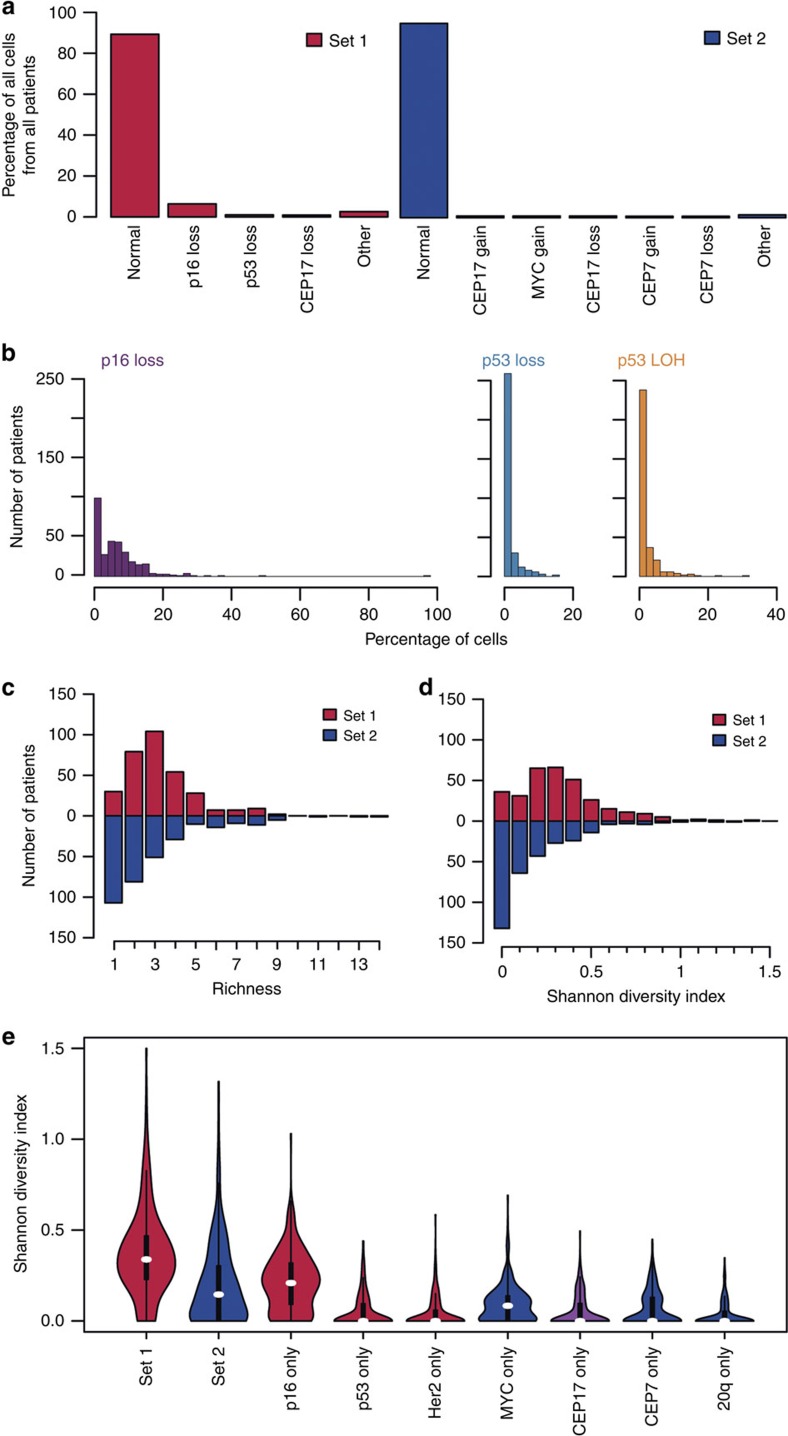
Patient properties. (**a**) Genotypes encountered in more than 0.5% of all scored. (**b**) Per cell per patient distributions of *p16* loss, *p53* loss and *p53* LOH. (**c**) Distribution of the number of different genotypes per patient. (**d**) Distribution of Shannon diversity indices per patient. (**e**) Distribution of Shannon diversity indices for whole-set and single-probe measures, colour-coded per probe set (red for set 1, blue for set 2, purple for both). For each violin plot, white marks define the median of each distribution, black rectangles delimit the second and third quartiles and vertical lines indicate the 95% confidence intervals while coloured shapes show the kernel density.

The majority of cells analysed had no detectable abnormalities: 89 and 95% of all cells analysed from probe sets 1 and 2, respectively, had wild-type genotypes (Fig. 1a). Across patients, the loss of one *p16* allele (hemizygous loss of *p16*) was the most frequently observed alteration overall with 51% of the patients (*n*=163) showing *p16* loss in at least 5% of cells (Fig. 1b; Supplementary Fig. 3). In contrast, *p53* loss observed in at least 5% of the cells was found in only 7.5% of patients (*n*=24), and relative *p53* locus loss^16^ (defined by copy-loss or fewer copies of the *p53* locus relative to the chr17 centromere (Methods)—observed in more than 5% of cells was seen in 10.6% of patients (*n*=34). In addition, comparison of the data from multiple brushes collected at the same endoscopy (termed repeat brushes) validated the reproducibility of our FISH analysis (Methods and Supplementary Fig. 4).

### A dynamic equilibrium of clones and evolutionary stasis

Previous studies of the clonal composition of individual Barrett's lesions have revealed genetic mosaicism^17^,^18^,^19^ and we sought to measure the degree of within-BE segment heterogeneity in our cohort. We defined a ‘clone' as the collection of cells with identical genotype (for example, identical copy-number of each of the four probes scored in the cells) and quantified genetic diversity using ecological diversity measures (Methods). Most samples contained multiple clones (*n*=290, 91.6% by probe set 1; *n*=213, 66.6% by probe set 2), and the distributions of diversity revealed a long tail of patients with very clonally diverse lesions (Fig. 1c,d). The genetic diversity measured using set 1 probes was significantly higher than for set 2 (*P*<0.001, paired *t*-test), owing to the prominence of *p16* and *p53* abnormalities assayed in set 1. We also evaluated the contribution of each individual probe to the overall level of diversity in the sample (Fig. 1e). Abnormalities of *p16* alone were as diverse as the whole second probe set (*P*=0.31, paired *t*-test). *p16* abnormalities explained the majority of the variance in clonal composition in set 1 (*R*^2^=0.74; *P*<2.2*e*^−16^), and *MYC* abnormalities were responsible for the majority of the genetic diversity in set 2 (*R*^2^=0.80; *P*<2.2*e*^−16^).

**Figure 2:**
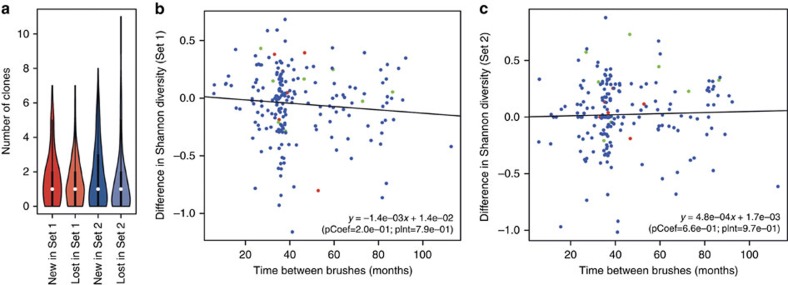
Genetic evolution of Barrett's oesophagus between endoscopic brushes. (**a**) Number of disappearing and appearing clones in both probe sets between time points (brushes). White marks define the median of each distribution, black rectangles delimit the second and third quartiles and vertical lines indicate the 95% confidence intervals while coloured shapes show the kernel density. Thick black lines indicate the middle quartiles and white dots highlight the medians. (**b**,**c**) Linear models of genetic diversity fitted to the time between time points. Blue dots: non-progressors; red dots: patients progressing after second time point; green dots: patients progressing before second time point.

We examined clonal evolution over time in the Barrett's patients by comparing the clonal composition between the two time points for the 195 patients where longitudinal data were available. An average of 1.4 (s.d. ±1.4) clones in probe set 1 and 1.4 (s.d. ±1.9) clones in probe set 2 detected at the first time point could not be detected at the second time point (termed ‘lost' clones), while 1.7 (s.d. ±1.6) clones in probe set 1 and 1.8 (s.d. ±1.8) clones in probe set 2 were found at the second time point but not the first (termed ‘new' clones) (Fig. 2a). However, bootstrap analysis suggested that nearly all of the observed slight changes in diversity could be reasonably explained by cell sampling, rather than *bona fide* changes in the clonal composition of the lesion (Supplementary Table 1). Nevertheless, a total of 32 significant changes in diversity were observed (16.4%; 29 non-progressors and 3 progressors) implying a dynamic turnover of clones within the lesion, whereby clonal expansions are balanced by the contraction of other clones. Further evidence of this ‘dynamic equilibrium' was provided by the observation of relatively stable levels of genetic diversity over time: the evolution of diversity did not show any significant trend over the follow-up interval for either probe set (Fig. 2b,c) and irrespective of the diversity measure used (Supplementary Fig. 5). Stratification of the cohort into non-progressors and progressors revealed that Shannon diversity decreased slightly over time for non-progressors only when measured by probe set 1 (*P*=0.012, paired *t*-test; Supplementary Fig. 6) but no significant difference was seen for non-progressors with probe set 2. Progression was associated with a slight increase in diversity over time in both probe sets, but was only statistically significant for samples having already progressed at the second time point in probe set 2 (*P*=0.03, paired *t*-test; Supplementary Fig. 6). We note that in 8 out of 14 of the eventual progressors, progression to HGD or EAC had already occurred at the time of the second brushing and that brushes were obtained prior to therapy (for example, ablation or endoscopic resection). Together, these data suggest that clonal structure of sizeable (and hence reliably detected) clones within non-dysplastic Barrett's lesions rarely change significantly over time. In other words, significant clonal evolution over large swathes of the Barrett's epithelium cannot be observed in the majority of Barrett's patients, but there rather is a ‘dynamic equilibrium' of clonal composition.

### Clonal expansions are rare

**Figure 3:**
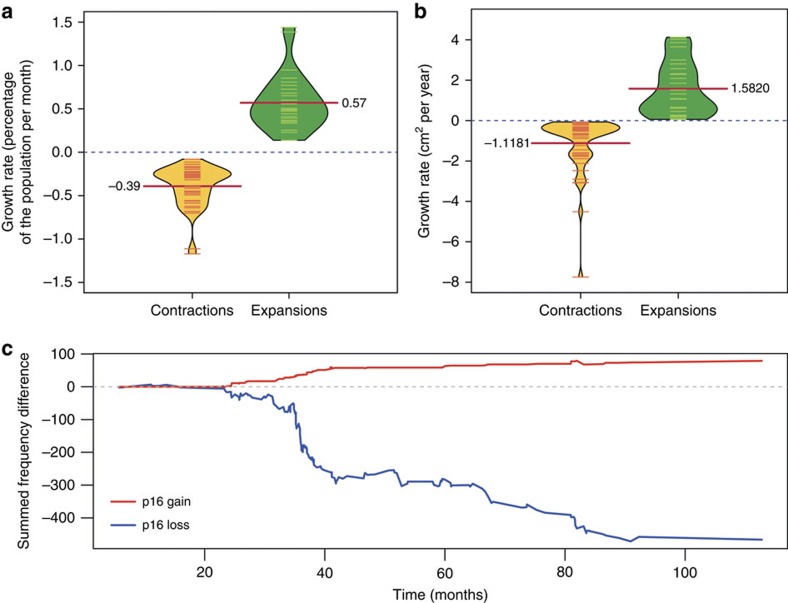
Clonal contractions and expansions. (**a**) Growth rate as the decrease/increase of each significant clonal expansion in percentage of the total cell population per month. (**b**) Growth rate as the decrease/increase of each significant clonal expansion in cm^2^ per year. Dashes are individual measurements; red lines annotated with a number represent the mean. (**c**) Evolution of *p16* loss and gain frequencies over time. The increase and decrease in frequency for both p16 loss (blue) and *p16* gain (red) is recorded at every second time point for all samples with multiple brushes to generate cumulative curves.

After correction for multiple testing, binomial analysis revealed 27 statistically significant clonal expansions and 45 clonal contractions (Supplementary Table 2) over 993.6 patient years of observation, an average of 1 detectable clonal expansion by these methods every 36.8 patient years and 1 clonal contraction every 22.1 patient years. The most frequent expansions and contractions over time were: normal genotype increase (*n*=23), normal genotype decrease (*n*=23) and *p16* loss decrease (*n*=19). Of the 32 cases of significant changes in diversity that we detected, 28 corresponded to significant clonal expansions/contractions, the 4 others being borderline significant for clonal expansions/contractions. We used the available data to try to approximate the growth rate of these clones within the Barrett's segment (Methods). Assuming linear growth, this translated to an average growth rate of 0.6% of the total cell population per month. Using 1.2 cm as the average radius of the oesophagus, this corresponds to an increase in the area of the Barrett's mucosa occupied by a growing clone of 1.58 cm^2^ (95% CI: 0.09–4.06) per year, while large contractions occurred at a rate of 0.4% of the cell population per month, with a corresponding decline in mucosal area of 1.12 cm^2^ (95% CI: 0.77–4.37) per year (Fig. 3a,b). Clonal expansions were not associated with progression (Fisher's exact test *P*=0.54).

### Clonal evolution of single abnormalities

We examined whether the individual abnormalities, as opposed to clones defined by all the loci in a probe set, underwent consistent (albeit slight) expansion or contraction over time. Only *p16* abnormalities and 20q loss showed consistent changes in clone size across patients (Supplementary Fig. 7a). Clones with loss of 20q tended to be larger at the second time point (*P*=3.5*e*^−5^, paired *t*-test) but this was not associated with progression (*P*=0.80, Cox proportional hazards model). Both contraction of clones with *p16* loss and expansion of clones with *p16* gain were observed (*P*=8.4*e*^−3^ and *P*=8.5*e*^−4^, respectively, *t*-test corrected for multiple testing). Interestingly, the contraction of clones with copy loss of *p16* appeared to be compensated by the expansion of clones with gains at the *p16* locus (Fig. 3c), suggesting that the former may be replaced by the latter over time (Supplementary Fig. 7b). Clones with a normal genome were also likely replacements for those having lost *p16* in most cases (Supplementary Fig. 7c), however, we note that we could not detect true copy-neutral loss of heterozygosity (cnLOH). The *p16* locus is assayed in our probe set 1, so these data partly explain the previously described lower diversity observed in set 1 values at the second time point. The expansion and contractions of *p16* mutant clones were not associated with progression (*P*=0.73 and *P*=0.55, respectively, Cox proportional hazards models).

### Analysis at a third time point

An additional third brush was available for a subset of 88 patients, including 5 progressors, taken at a median of 36 months (range 13–93 months) after their second brush and at a median of 72 months after the first brush (range 45–143). These brushes surprisingly revealed a general decrease in diversity in set 1, but no difference in diversity in set 2 (Supplementary Fig. 8). Analysis of clonal frequencies revealed that *p16*-loss clones tended to have contracted or even disappeared at this time point (*P*<0.001, Supplementary Fig. 9), similar to what we observed between the first two time points. Of the 88 patients with a third brush, 22 showed a significant contraction of *p16*-loss clones between the first and third time points, 3 showed a significant expansion (Supplementary Table 3). Moreover, expansions or contractions of clones with abnormal *p16* copy number accounted for all significant changes in diversity in set 1 (Supplementary Fig. 8, right column). In addition, the five progressors appeared to have moderately higher diversity values than non-progressors, although this was rarely significant (Supplementary Fig. 10). The rate of clonal contractions of *p16*-loss clones between the second and third time points was similar to the rate between the first and second time points (Wilcox-test *P*=0.13; Supplementary Fig. 11), although affecting entirely different patients, despite the less stringent multiple correction for this smaller cohort at the third time point, which facilitates detection of smaller changes in clone frequencies. Similarly to our observations between earlier time points, clones appeared to be gained at similar rates (1.7±1.1 clone appearing and 1.6±1.6 clone disappearing between the second and third time points in set 1; 1.5±1.1 appearing and 1.6±1.8 disappearing in set 2). Together, these data suggest a general and steady clonal contraction of *p16*-loss clones over time in the Barrett's segment that was not mirrored by any other locus assayed.

### Diversity measures determine progression risk

Our previous work has shown that a high level of clonal diversity (measured using biopsies and microsatellite LOH assays) within a Barrett's segment is associated with increased risk of progression to cancer^13^,^15^. In this independent cohort of only non-dysplastic Barrett's patients, we validated these previous observations using a different genetic assay and whole surface brushings instead of random biopsies. Higher levels of clonal diversity that now were measured by FISH on endoscopic brushings were associated with increased risk of progression to HGD or cancer, this result being largely robust to the choice of diversity statistics, and the FISH probes included in the measure; univariate Cox proportional hazards analysis showed that out of the nine statistically significant predictors of progression, seven were diversity-based, and the two others being aneusomy and age (respectively seventh and eighth most significant) (Table 2). Surprisingly, the size of the biggest clone, evidenced by *p53* loss and/or *p53* relative locus loss was not a prognostic marker. Thus, in this cohort of BE patients with no dysplasia, *p53* loss did not bear prognostic potential, which is in contrast to findings in Barrett cohorts that also included patients with low- and high-grade dysplasia^16^,^20^,^21^,^22^. We included the significant prognostic markers in a multivariate model with age and circumferential Barrett's length (accepted prognostic factors^23^,^24^,^25^) and found that all eight genetic variables were the most significant predictors of progression (Supplementary Table 4). The segment length was not a significant prognosticator and we note that while several studies have indicated that there is an increased risk in longer segments, others were not able to confirm this finding^26^,^27^.

**Figure 4:**
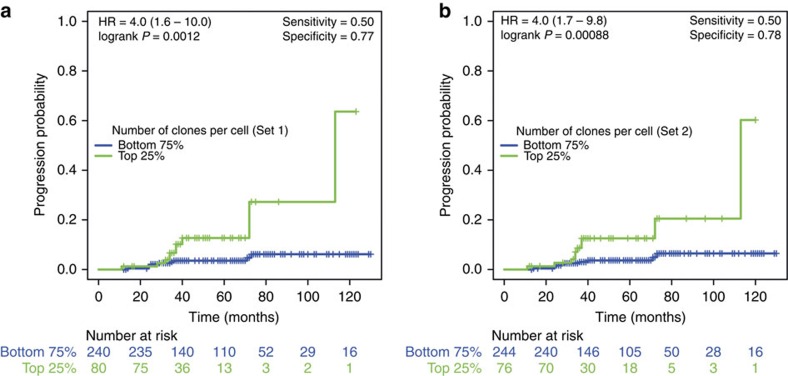
Examples of Kaplan–Meier curves for genetic diversity-based patient stratification. Coloured lines indicate the proportion of patients progressing to cancer and vertical bars indicate right censoring (end of follow-up data). (**a**) Stratification based on the number of clones per cell in the first probe set. (**b**) Stratification based on the number of clones per cell in the second probe set.

Interestingly, two of the best performing variables were obtained by single-probe diversity measures that used just the CEP7 and *MYC* probes, respectively. We stratified patients as high- and low-risk using two different thresholds for each significant diversity measure: (1) samples with values higher than median (>median threshold) being high risk; (2) samples with values in the upper quartile (top 25% threshold, used in previous studies^13^,^15^) being high risk. We found that the genetic diversity measures consistently and effectively separated the progressors and non-progressors into the appropriate risk groups (Fig. 4, Supplementary Fig. 12 and Supplementary Table 5). These observations suggest that the level of clonal diversity determines the progression risk in non-dysplastic Barrett's patients and that this risk can even be assessed using single-probe clonal diversity measures of *MYC* or CEP7.

We note that neither the size of the clone(s) with *p16* copy-number alterations nor the diversity of *p16*-altered clones were significantly associated with cancer development risk (Table 2). Thus, while *p16*-altered clones show interesting clonal dynamics over time, these dynamics appear not to be directly related to the cancer development risk.

### Progression risk is invariant over time

Our data indicated that the level of clonal diversity measured at baseline in non-dysplastic BE was indicative of progression risk, and that this level of diversity did not change significantly over time. Consequently, we hypothesized that the risk of progression to HGD or cancer was established early in the development of a Barrett's lesion and remained invariant thereafter. To test this idea, we re-classified patients as high- or low-risk using diversity measures obtained from the second time point (without changing the previously defined risk-stratification cutoffs) and tested whether these later diversity measures were predictive of the initial progression risk. All measures remained significant predictors of progression, with the exception of age (Supplementary Fig. 13, Supplementary Table 6) and 10 out of 18 stratifications were still significant after multiple testing. The average diversity of the two time points was similarly predictive. We further reproduced this analysis after removing the eight patients that had already progressed to HGD or cancer at the second time point, leaving only six progressors. This hindered statistical power but the Shannon and Simpson diversity indices were still significant (Supplementary Fig. 14). We performed a bootstrap analysis to test whether cell sampling error could lead to patients being moved from low- to high-risk groups or vice-versa. Our analysis revealed that the best diversity-based stratifications correctly identified all but one progressor in 80–100% of all bootstrap simulations (Supplementary Fig. 15). Therefore, the time at which the BE segment is analysed appears to have little impact on our ability to distinguish high- and low-risk patients, suggesting that patients' risk of progression remained constant over time. Taken together, we found that the progression risk of non-dysplastic patients can be effectively assessed by clonal diversity measures, and that this predetermined risk is stable over time. The few progressors for which we had a brush from a third time point (*n*=5) prevented the reproduction of this analysis at this time point.

## Discussion

We have performed a longitudinal study of clonal evolution in a cohort of non-dysplastic BE at single-cell resolution. At this cellular resolution, we have been able to confirm previous reports of frequent and extensive clonal mosaicism within the Barrett's segment^17^,^19^ in cells collected evenly across the whole Barrett's segment, and validated that genetic diversity is a powerful predictor of progression insensitive to the choice of diversity statistics using^13^. Although clonal expansions in our patients were generally rare, we provide quantification of the frequency and rate of clonal expansions in a human neoplasm. We only observed one significant clonal expansion every 36.8 patient years of follow-up, and in those cases, the clones grew at an average of 1.58 cm^2^ per year.

Importantly, our data show that measures of clonal diversity are more prognostic than ‘traditional biomarkers' that are based on the detection of particular individual genetic abnormalities. The relative utility of diversity measures may be due to a number of factors, not least that traditional biomarkers are prone to sampling errors, for instance when assayed in randomly taken biopsies, or when neoplastic progression proceeds along a non-assayed pathway. In contrast, diversity measures exploit lesion heterogeneity as a proxy measure of evolvability, the idea being that more diverse lesions are more likely to contain or produce a ‘well-adapted' clone that is able to drive carcinogenesis. We note that diversity of specific markers was more prognostic than others, and so optimal prognostication will benefit from careful choice of markers. Most strikingly, we demonstrate that several of the single-probe diversity measures (*MYC* and *CEP 7*) were the best predictors in the multivariate analysis, and conversely that *p16*-abnormalities are poor prognosticators due to the initial expansion and then contraction of those clones. Together our data underline the potential for clonal diversity measures as robust biomarkers for cancer risk stratification in endoscopically surveyed Barrett's patients.

Our results are consistent with previous studies of the Seattle Barrett's oesophagus cohort^28^. That cohort, which includes dysplastic as well as non-dysplastic BE, has been characterized by single-nucleotide polymorphism arrays applied to the purified epithelium from whole biopsies (one every 2 cm of the BE segment). The degree of genetic divergence between biopsies remained relatively constant in the non-progressors but 24–48 months prior to progression, massively genetically altered clones started to appear in the progressors. Also in the Seattle cohort, clones did not tend to sweep to fixation, and the resulting higher levels of divergence remained stable over time. Importantly, in this study we have provided a view of the spatial and temporal dynamics of clones at cellular resolution.

**Figure 5:**
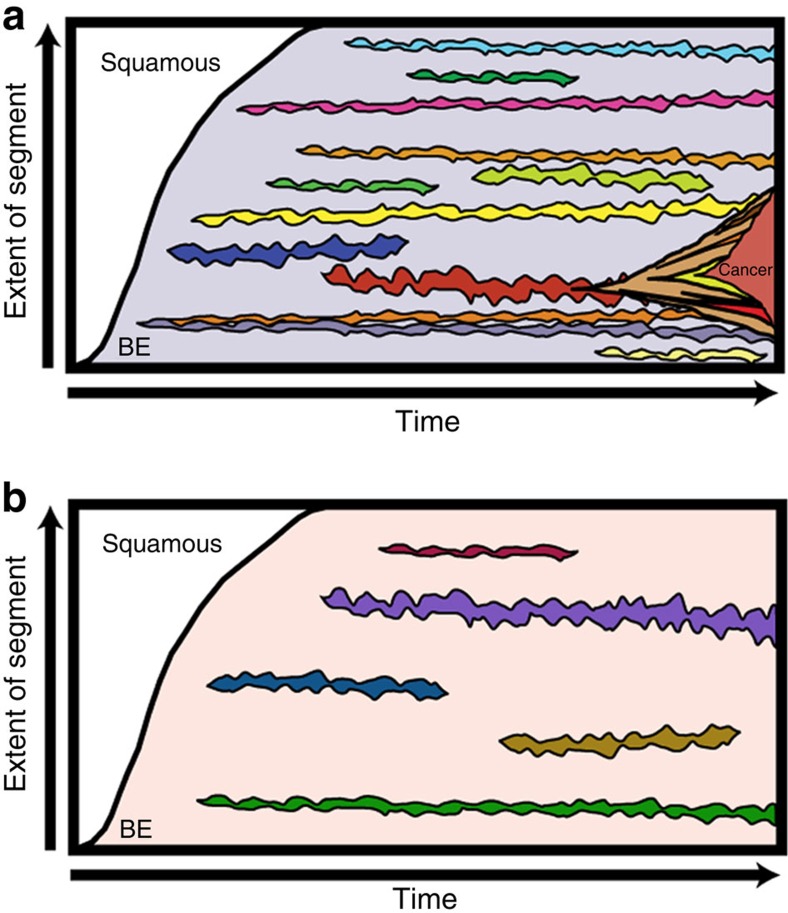
Updated evolutionary model of Barrett's oesophagus. Clones can appear without leading to major clonal sweeps in the population, eventually dying out or leading to cancer progression. (**a**) Sample with stable high genetic diversity. (**b**) Sample with stable low genetic diversity.

Due to (likely) lead time bias, the highest incidence of progression of Barrett's patients is during the first year of surveillance whereas the progression risk of the remaining non-dysplastic Barrett's surveillance cohort is low. High level evidence from prospective studies of robust biomarkers to distinguish future progressors from non progressors in these low risk cohorts are lacking^22^. In line with the clinical observation that progression is rare is the fact that we observed minimal evolution occurring in our non-dysplastic Barrett's cohort over the duration of surveillance. Clonal stasis has been previously suspected^14^,^28^, but the sparse biopsy sampling used in past studies (as opposed to the endoscopic brushes used here) has meant that moderately sized clonal expansions may have gone undetected. The norm appears to be that once a dynamic equilibrium of clones in the non-dysplastic Barrett's lesion is established clonal diversity levels remain relatively constant thereafter. Consequently, those lesions that are established with a high level of clonal diversity appear to be inherently prone to cancer development, whereas lesions established with a low level of clonal diversity appear to be intrinsically non-progressive. Our data provide some evidence (within the limits of our lesion sampling) of a continual dynamic turnover of clones within the Barrett's mucosa such that the equilibrium level of diversity is likely to be maintained by an ever changing mosaic of clones. It is, however, not clear at which time point the dynamic equilibrium in the Barrett's tissue is reached. One known risk factor for progression of Barrett's is active reflux disease^29^. In all study participants, reflux control was achieved through effective acid suppressive therapy prior to inclusion and so it remains to be studied how clones and diversity evolve in a Barrett's patient with active reflux and esophagitis, and correspondingly whether or not diversity reaches the stable equilibrium after effective treatment with acid-suppressive therapy. An observation in this study was the slight decline in diversity and contraction of clones over time that was attributable to the decrease in the size of clones that had lost a copy of the p16-locus. Consequently, we speculate that loss of the p16-locus provides an advantage to clones experiencing reflux, but a disadvantage once the acid is suppressed (and so possibly clones that undergo homologous recombination—which were undetectable in our study and could involve duplicating an inactivated *p16* copy—may experience positive selection). The trend towards ‘genetic normalization' could further be due to the (microscopic) mucosal healing associated with the effects of longstanding anti-reflux therapy as is achieved during follow-up and management of patients in surveillance programs. This perhaps explains the beneficial effects of periodic surveillance on patient compliance to therapy and thus the progression risk, which tends to be lower than expected in surveyed cohorts. Moreover, this line of reasoning highlights that the evolutionary dynamics (and indeed genetic markers thereof) may be different in patients who receive acid-suppressive therapy compared with those who do not. Nevertheless, our data suggest that an increased risk of progression of a Barrett's lesion is determined early (Fig. 5). The emergence of altered clones 24–48 months prior to the progression in the Seattle cohort^28^ is comparable to the high diversity status of future progressors and the average progression interval in our cohort, and therefore it is possible that cancer risk is mediated by the acquisition of genetic instability early in lesion development. Longer follow-up is required to confirm very-long-term evolutionary stasis and invariance of cancer risk.

Our observation has obvious and important consequences for clinical surveillance of the large cohorts of low-risk Barrett's patients; principally that an increased risk of cancer is invariant over a period of at least 3–4 years and can be determined from a single diversity measurement using specific markers. Our data shed light on the evolutionary dynamics of progression to cancer in BE. Rather than the previous stepwise-model of sequential clonal expansions that sweep to fixation, driving other clones extinct^30^, our data support the co-existence of multiple clones^17^ in which new clones are regularly spawned but undergo minimal clonal expansion, and often go extinct (Fig. 5). The *p16* locus is frequently involved in the rare large clonal expansions that do occur, suggesting a special role for this locus in disease aetiology, but was not found to predict cancer progression. Indeed, our results suggest that among the seven markers we examined, only losses at the *p16* locus experienced consistent clonal selection. Only when the correct combination of genetic events occurs will a cancer develop: the lack of prior clonal sweeps implies that progression may be a ‘punctuated' event that requires the ‘lucky' acquisition of a complete set of genetic changes by a single (small) clone, either in one catastrophic event or sequentially, in an otherwise evolutionary quasi-static cell population.

Our longitudinal study reveals a new picture of the dynamics of carcinogenesis in BE where the clonal make-up and evolutionary trajectory of the lesion is predetermined from the outset. Recognizing that only a subset of non-dysplastic Barrett's are ‘born to be bad' offers new hope for effective risk stratification of this challenging patient group. These novel measures need to be based on universal biomarkers measuring the evolutionary dynamics of neoplasms.

## Methods

### Patient data

Between 2002 and 2013, we performed a prospective cohort study in which patients were recruited from one academic medical centre and six general hospitals in the Netherlands. Criteria for the inclusion were: (a) age >18 years, (b) endoscopic evidence of BE, (c) presence of specialized intestinal metaplasia without dysplasia in biopsies of the baseline endoscopy, (d) no history of HGD or EAC or prior endoscopic therapy for BE and (e) no endoscopic features of active reflux esophagitis. All patients that developed dysplasia or EAC within 6 months from the index endoscopy were excluded from the study. The Medical Ethics Committee of the Academic Medical Center, Amsterdam approved the study and all patients provided written informed consent. All patients underwent endoscopic surveillance every 2–3 years in adherence to the international guidelines^6^,^24^.

Endoscopic findings from all participants were registered in a central database along with the endoscopic and pathology reports, and clinical data including sex, age, circumferential Barrett's segment length, BMI, use of proton-pump inhibitors, family history of BE and/or EAC, and smoking. During the baseline and subsequent follow-up endoscopies, a cytology specimen was obtained from the Barrett's mucosa for genetic evaluation by a single-cell-based analysis using DNA FISH. Patients were considered progressors when they developed HGD or EAC during follow-up. Follow-up time was defined as the time from the baseline endoscopy to the date of the most recent surveillance endoscopy or to the date of the endoscopy based on which patients were diagnosed with HGD or EAC. Patient data are summarized in Supplementary Data 1.

### Brushes and biopsies

Samples for FISH analysis were taken from the whole Barrett's segment using a standard endoscopic cytology brush (Cook Endoscopy, Winston-Salem, NC). Prior to brushing, a mucolytic agent (acetylcysteine, 50 mg ml^−1^) was applied to dissolve the mucus layer. Brushes were stored in PreservCyt solution (Hologic, Marlborough, MA) and concentrated in 3 ml by removal of the supernatant after centrifugation. Following the cytospin procedure (Shandon Cytospin 4, Cytocentrifuge, Thermo, Waltham, MA), the cells were concentrated on a slide in a uniform monolayer and stored at −80 °C until further processing for FISH analysis.

Biopsies were then taken for pathological evaluation following the Seattle protocol (four-quadrant biopsies every 2 cm, together with the targeted biopsies of any visible lesions). Each biopsy was evaluated for the degree of dysplasia according to the Vienna classification^31^. Cases of dysplasia or EAC reported by the local pathologist were reviewed by two pathologists who were part of a central expert pathology panel.

### FISH

We selected seven markers, split in two panels of four markers, out of 12 from a larger panel based on the existing literature on biomarkers on Barrett's, including karyotyping, CGH, LOH data^32^. FISH was performed using fluorescent locus-specific and chromosomal centromeric (CEP) probes (Abbott Molecular). Chromosomal abnormalities were assessed in two different probe sets with set 1 comprising CEP17, *ERBB2* or *Her-2/neu* (17q11.2-12), *p53* (17p13.1) and *p16* (9p21) and set 2 comprising CEP7, CEP17, 20q (20q13.2) and *MYC* (8q24.12). A fluorescent microscope (Olympus BX61) equipped with specific band filters was used to evaluate the slides. Signal patterns were recorded for consecutive interphase nuclei of non-squamous, non-inflammatory cells (attempted to count 100 cells per probe set). For each individual cell the copy number of each of the four loci was recorded by manual counting by an experienced FISH technician (C.T.L.) who was blinded to the clinical and histological findings.

A second brushing was performed in three patients, using a new clean brush, immediately after the first brush was taken. FISH analysis was performed as per all other brushes. To check for consistency in the FISH signals, the diversity from set 1 and set 2 measured in the first brush was compared with the second brush using the resampling method described in the ‘Sampling Bias Analysis' section below. All single-cell FISH measurements are reported in Supplementary Data 2 and illustrative pictures are shown in Supplementary Fig. 16.

### Minimum cell number

To determine the minimum acceptable number of cells to accurately describe per-sample statistics (for example, a clonal diversity measure) we performed a bootstrap simulation. 1,000 random subsamples of size *n*_s_ cells (possible *n*_s_ values: 10, 20, 30, 40, 50, 60, 70, 80, 90) were made by sampling without replacement from the total of *n*_t_ cells. We then, for each statistic evaluated, we calculated the mean and s.d. across the 1,000 bootstrap replicates as a function of the sample size *n*_s_. The variance of most statistics stabilized when 50 cells or more were included and we thus analysed only the samples with at least 50 scored cells (Supplementary Fig. 1).

### Prognostic markers

We evaluated ‘individual markers' as the percentage of analysed cells with an analysis were expressed as the percentage of cells in a sample representing the individual genetic abnormalities, which included *p53* loss, *p16* loss, *Her-2/neu* gain, 20q gain, *MYC* gain and aneusomy (defined by the mean of the percentage of cells with abnormal CEP17 in both sets and the percentage of cells with abnormal CEP7).

To evaluate per cell variables; *p53* LOH was defined as cells in which the copy number of the *p53* locus was inferior to 2 or to the copy-number of centromere 17. The biggest clone size was defined by multiplying the frequency of the most prominent non-normal genotype (for example, max (*p*_*i*_)) by the circumferential (*C*) length of the BE segment.

Diversity measures were analysed to quantify clonal diversity within the Barrett's segment. Clones were defined as the collection of cells with the same genotype, itself defined by the combination of probe-specific copy numbers. The number of cells observed in a particular sample was denoted as *N*, the number of genotypes (different clones) observed in the sample as *R*, and their frequency within the sample as the set {*p*_*i*_}, and the genotype of *k*^th^ locus in the *i*^th^ cell as *a*_*ik*_.

The Shannon and Simpson diversity indices take into account the number of different clones as well as the abundance of each clone.

The Shannon index is given by:


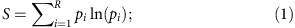


The Simpson index, which places a greater weight on the more abundant clones, is calculated as: 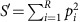;

The average pairwise genetic divergence is a distance metric and is qualitatively different from the Shannon, Simpson and number of clones statistics. Rather than measuring the number and abundance of clones, it estimates the time since cells shared a common ancestor, based on how much they have genetically diverged from each other. It is calculated as:


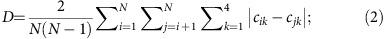


The number of different clones observed in a sample (*Nc*) was normalized to the total number of cells in the sample (*Nc*/*N*), since the more cells that are assayed, the greater the chance of observing a new clone. In contrast to Simpson's index, this statistic places a greater weight on the less abundant clones. In addition to the diversity measures computed on the genotypes defined by whole probe sets, we also calculated single-probe Shannon diversity indices, taking into account only a single locus at a time.

### Sampling bias analyses

We first proceeded to assess our ability to reliably observe large clonal expansions and contractions for all clones present at baseline in samples with multiple time points. For each clone *i* present in a sample *s*, we proceeded to a binomial test using the binom.test standard *R* function, defining the following: *p*_*i*_, the probability that any cell at time point 1 is of clone *i*; *x*_*i*_, the number of observations of *i* at the second time point; *n*_*s*_, the number of cells scored for *s* at the second time point.

The second resampling analysis concerned the relation between sampling bias and the measurement of diversity over time. For each sample with multiple time points, we pooled all cells scored at both time points. For each sample *s* and time point *t*, we drew *n*_*t*_, the number of scored cells for sample *s* at time point *t*, at random from the pool of clones and computed the resulting diversity measure (Shannon and Simpson indices, number of clones per cell, average pairwise divergence). The process was repeated 1,000 times to create a background genetic diversity distribution for each endoscopic brush. For each sample, we produced the distribution of pairwise differences between all resampled *t2* diversity indices minus all resampled *t1* indices. A *Z* score was then attributed to the difference in diversity observed in the real data compared with the resampled difference distribution; a resulting *P* value was computed assuming a normal distribution.

Finally, the resampled distribution of each diversity measure was used to assess the reliability of the diversity-based classifications presented in Supplementary Table 4. On the basis of the selected threshold for stratification (above median or top 25% of *t1* observation to define high risk), we computed the frequency at which each patient would switch category.

We analysed both probe sets with the same methodologies and used Bonferroni correction for multiple testing. For the sample that had both a pre- and post-progression time point, we used the pre-progression time point for both analyses.

### Rate of clonal expansion

For each clone *i* in a patient *p* with two time points, we defined the growth rate *g*_*i*_ and length-normalized growth *G*_*i*_ rate of all clonal expansions using the following formulas:


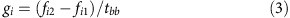



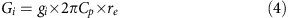


where *f*_*i*1_ is the frequency of clone *i* in patient *p* at the first time point, *f*_*i*2_ its frequency at the second time point, *t*_bb_ the time between the brushes and *C*_*p*_ the circumferential length of the Barrett's segment in patient *p*. *r*_e_ represents the average radius of the adult human oesophagus and was set to 1.2 cm.

Clonal expansion may only occur at the clone boundary meaning that the clone will grow quadratically. Therefore we have that:


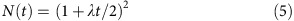


Where *N(t)* is the percentage size of the clone at time *t*, and we solved for the quadratic growth rate *λ* (% cells per year) using the clone abundances at the two time points (Supplementary Table 2).

### Data availability

The authors declare that all data is available within the Article and its Supplementary Information files, or available from the author upon request

## Additional information

**How to cite this article:** Martinez, P. *et al.* Dynamic clonal equilibrium and predetermined cancer risk in Barrett's oesophagus. *Nat. Commun.* 7:12158 doi: 10.1038/ncomms12158 (2016).

## Supplementary Material

Supplementary Information Supplementary Figures 1-16 and Supplementary Tables 1-6.

Supplementary Data 1 Marker scoring for all brushes.

Supplementary Data 2 Patient data.

## Figures and Tables

**Table 1:** Clinical characteristics.

Characteristics	Entire cohort	Patients with follow-up brush
Total number of patients	320	195
		
Male sex fraction	80.9% (*n*=259)	81.0% (*n*=158)
		
Average age	58.9±11.7	58.6±11.2
		
Median *C* length	2 (95% CI: 0–9)	2 (95% CI: 0–9)
		
Average BMI	27.2±3.9	27.1±3.7
		
Use of proton-pump inhibitors	99.7% (*n*=319)	99.5% (*n*=194)
		
Family history of Barrett's oesophagus	11.3% (*n*=36)	11.8% (*n*=23)
		
Family history of oesophageal cancer	8.8% (*n*=28)	7.7% (*n*=15)
		
Smoking	69.4% (*n*=222)	72.3% (*n*=141)
		
Progressors	6.3% (*n*=20)	7.2% (*n*=14)

**Table 2:** Individual prediction performance.

Variable	Unit	*P* value*	HR	95% CI
Number of clones per cell (set 2)	per %	***0.0037***	1.19	1.06–1.34
Centromere 7 only Shannon diversity	per 0.1	***0.0046***	1.72	1.18–2.50
Number of clones per cell (set 1)	per %	***0.005***	1.29	1.08–1.54
Avg. pairwise divergence (set 2)	per 1.0	***0.0088***	1.37	1.08–1.74
MYC only Shannon diversity	per 0.1	***0.0093***	1.67	1.13–2.46
Shannon diversity (set 2)	per 0.1	***0.013***	1.20	1.04–1.38
Aneusomy	per %	***0.016***	1.12	1.02–1.23
Age	per year	***0.017***	1.05	1.01–1.10
Simpson diversity (set 2)	per 0.1	***0.028***	1.51	1.05–2.17
Shannon diversity (set 1)	per 0.1	0.052	1.16	1.00–1.34
*C* length	per cm	0.062	1.12	0.99–1.26
*MYC* gain	per %	0.063	1.19	0.99–1.44
Chromosome 20q only Shannon diversity	per 0.1	0.075	1.60	0.95–2.70
Simpson diversity (set 1)	per 0.1	0.10	1.27	0.95–1.69
Avg. pairwise divergence (set 1)	per 0.1	0.11	1.17	0.96–1.41
Abnormal cell % (set 1)	per %	0.12	1.02	0.99–1.05
*p53* only Shannon diversity	per 0.1	0.12	1.33	0.93–1.89
Abnormal cell % (set 2)	per %	0.17	1.02	0.99–1.06
Centromere 17 only Shannon diversity	per 0.1	0.18	1.26	0.90–1.78
*p16* loss	per %	0.28	1.02	0.98–1.05
*Her-2* only Shannon diversity	per 0.1	0.29	1.23	0.84–1.81
*p53* LOH	per %	0.32	1.04	0.96–1.12
Chromosome 20q gain	per %	0.37	1.12	0.88–1.42
Biggest clone size	per cm	0.40	1.15	0.83–1.59
*p16* only Shannon diversity	per 0.1	0.47	1.10	0.85–1.41
*Her-2* gain	per %	0.57	1.03	0.92–1.16
*p53* loss	per %	0.71	1.03	0.89–1.18
BMI	per kg per m^2^	0.93	1.00	0.90–1.12

*P values obtained with univariate Cox proportional hazards models. Bold and italic values indicate statistical significance (*P*<0.05).
