# Supplementary material for: Dynamic clonal equilibrium and predetermined cancer risk in Barrett's oesophagus
Source: Nat Commun. 2016 Aug 19;7:12158. doi: 10.1038/ncomms12158 (PMC4992167; doi:10.1038/ncomms12158)
Supplement: Supplementary Information — Supplementary Figures 1-16 and Supplementary Tables 1-6. [file ncomms12158-s1.pdf]

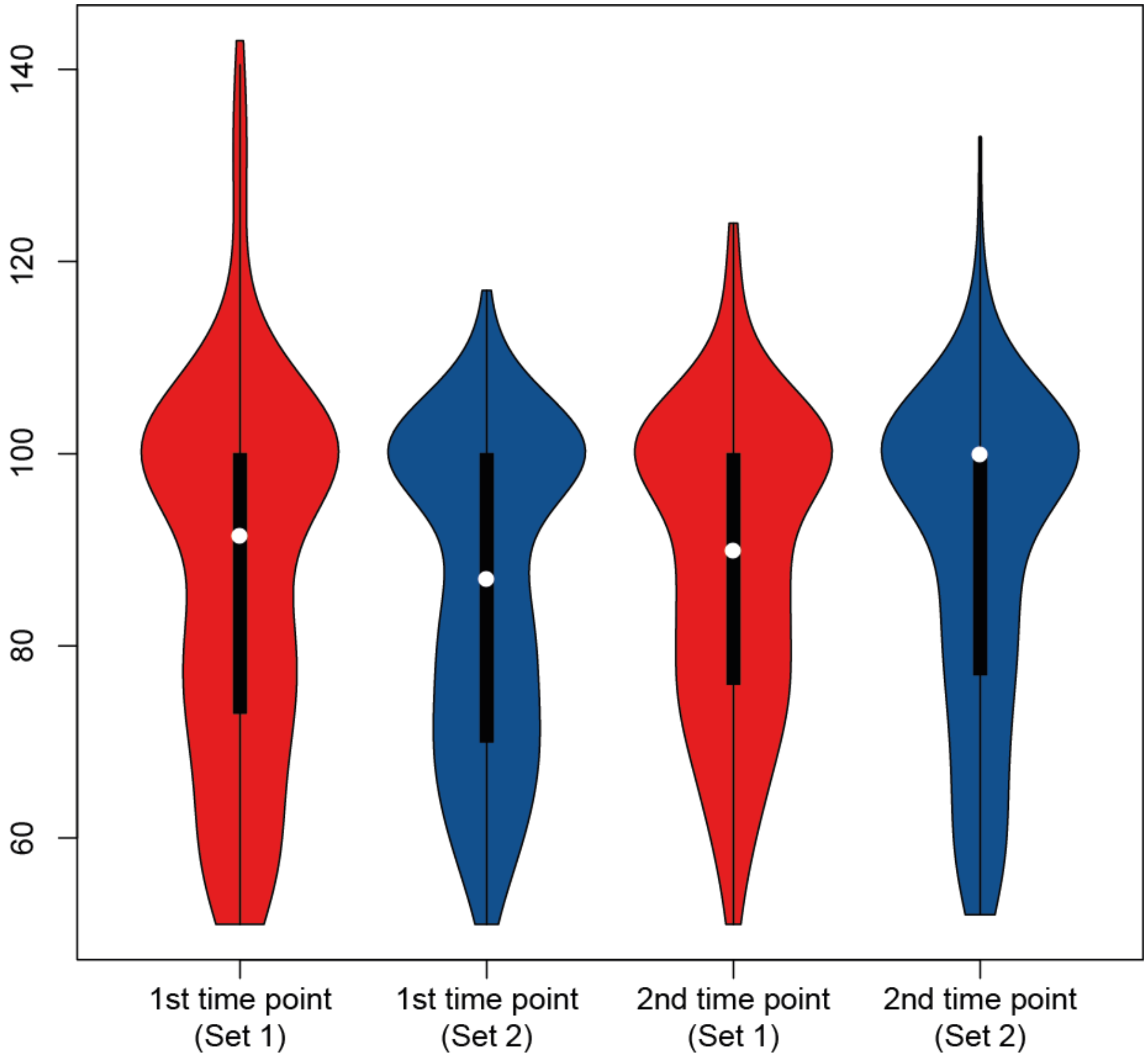

Supplementary Figure 1: Distribution of scored cells per brush for each time point and probe set. Beanplots highlight the distribution density (blue/red wave) with thick black lines highlighting the middle quartiles. Medians are represented by white dots. For each violin plot, white marks define the median of each distribution, black rectangles delimit the 2nd and 3rd quartiles and vertical lines indicate the 95% confidence intervals while colored shapes show the kernel density.

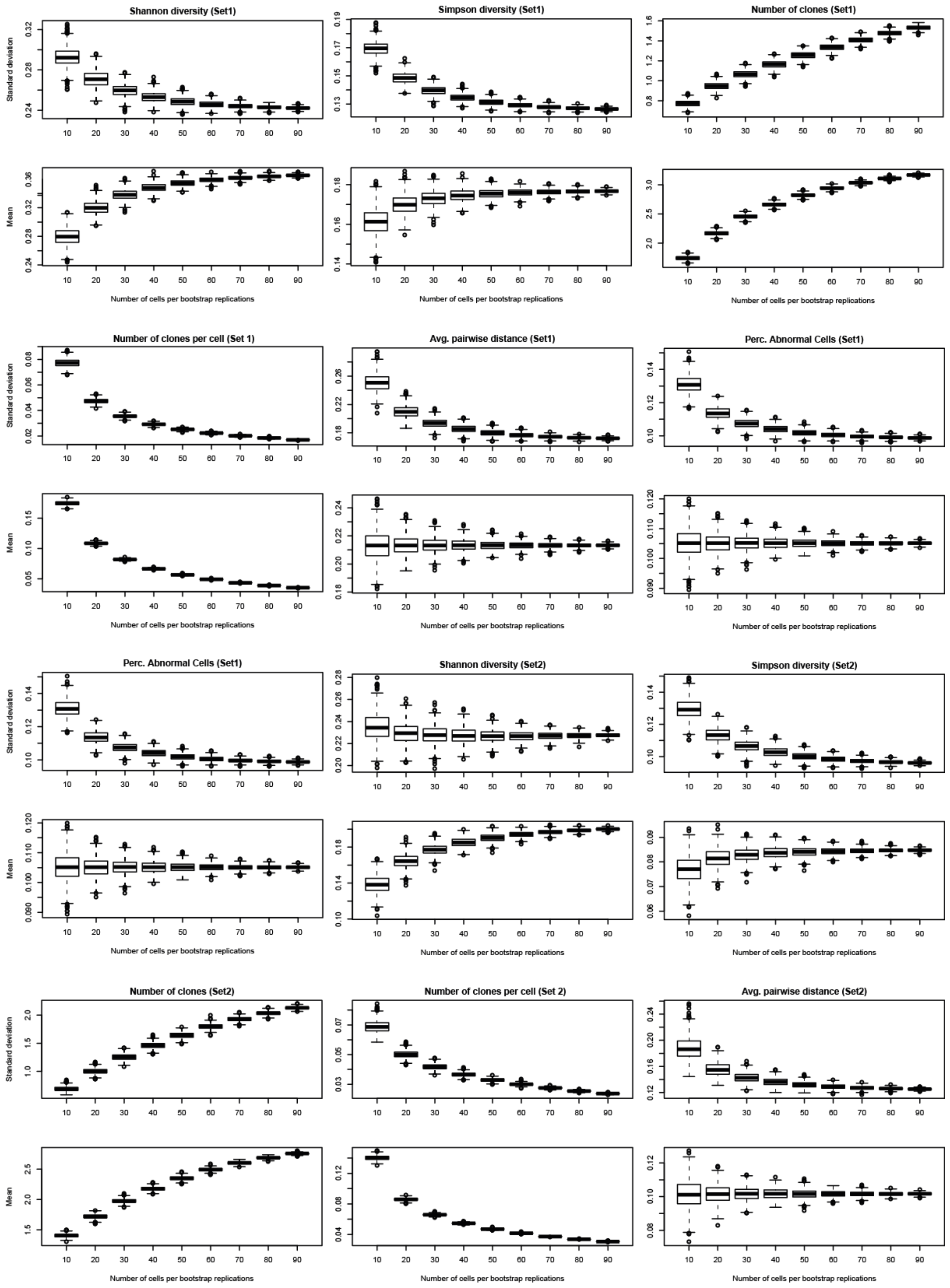
Shannon diversity (Set1)
Simpson diversity (Set1)
Number of clones (Set1)
Number of clones per cell (Set 1)
Avg. pairwise distance (Set1)
Perc. Abnormal Cells (Set1)
Perc. Abnormal Cells (Set1)
Shannon diversity (Set2)
Simpson diversity (Set2)
Number of clones (Set2)
Number of clones per cell (Set 2)
Avg. pairwise distance (Set2)
Standard deviation
Mean
Number of cells per bootstrap replications
10 20 30 40 50 60 70 80 90

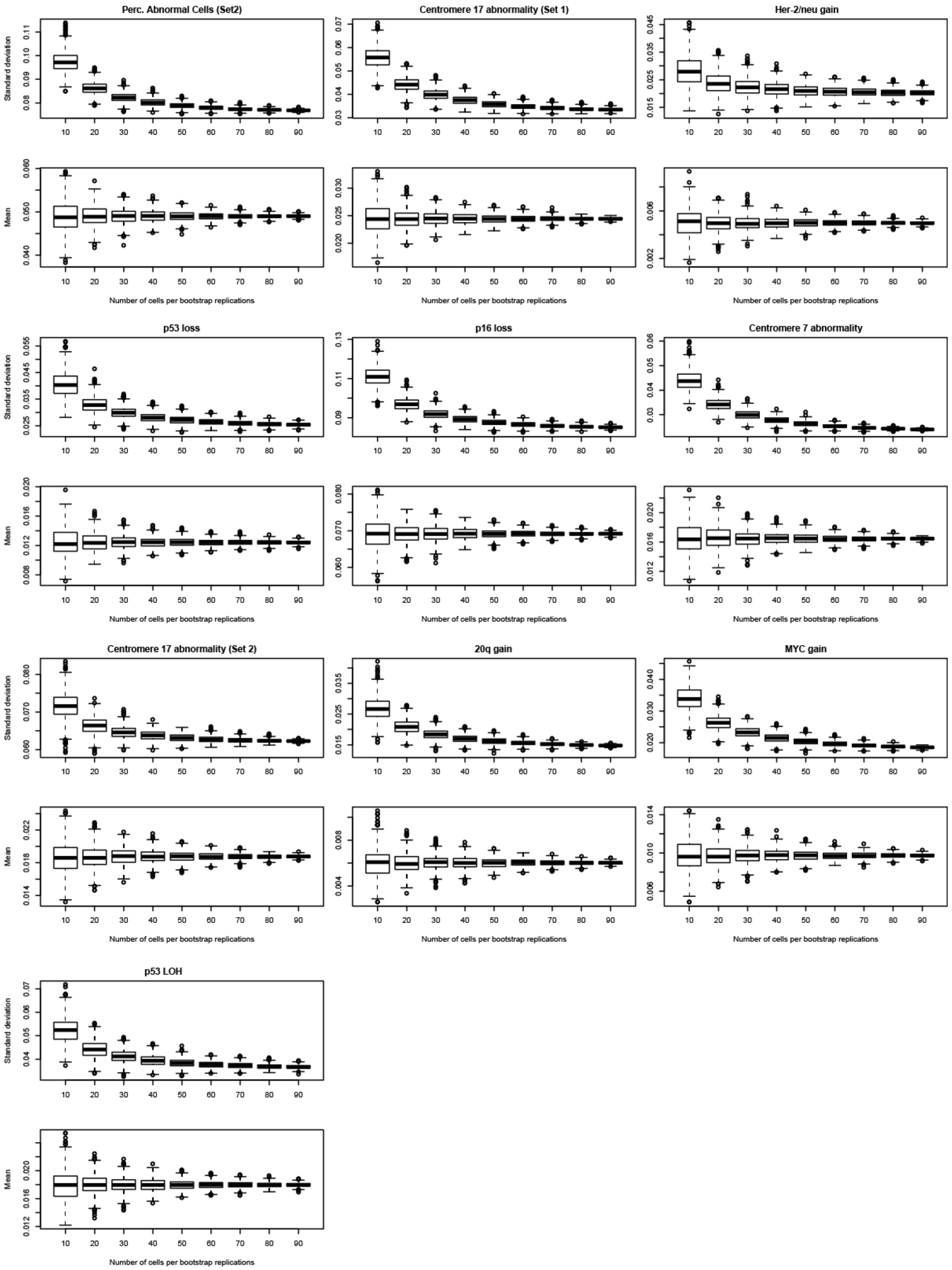

Supplementary Figure 2: Mean and standard deviation of all markers during bootstrapping. The bootstrapping procedures consisted in taking *N* cells from each sample and recomputing each marker. *N* varied from 10 to 90 in 10 cells increments. Boxes indicate the lower and upper boundaries of the 2nd and 3rd quartiles, whiskers indicate 95% confidence intervals, dots indicate outliers.

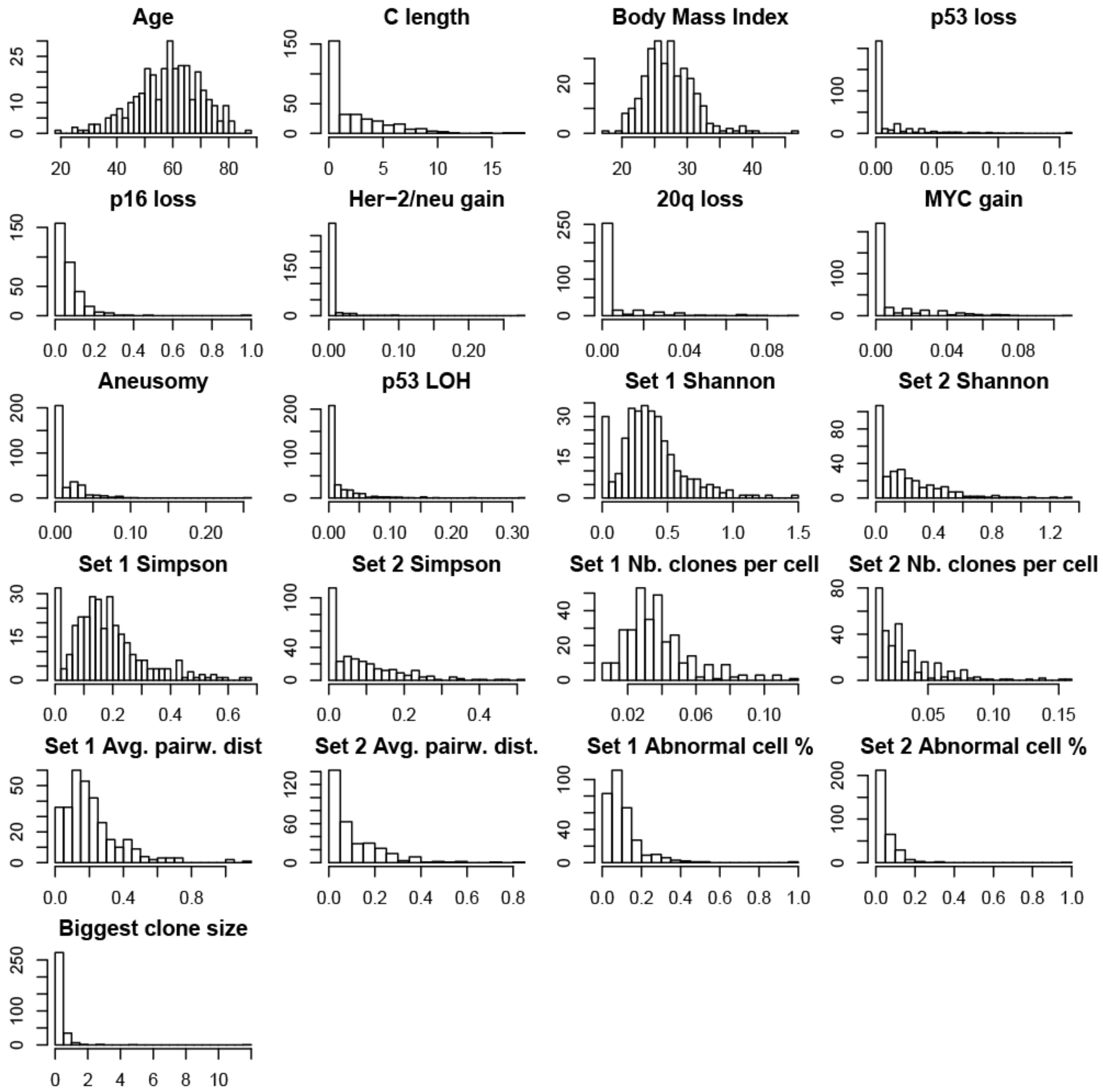

Supplementary Figure 3: Histograms for all markers at baseline.

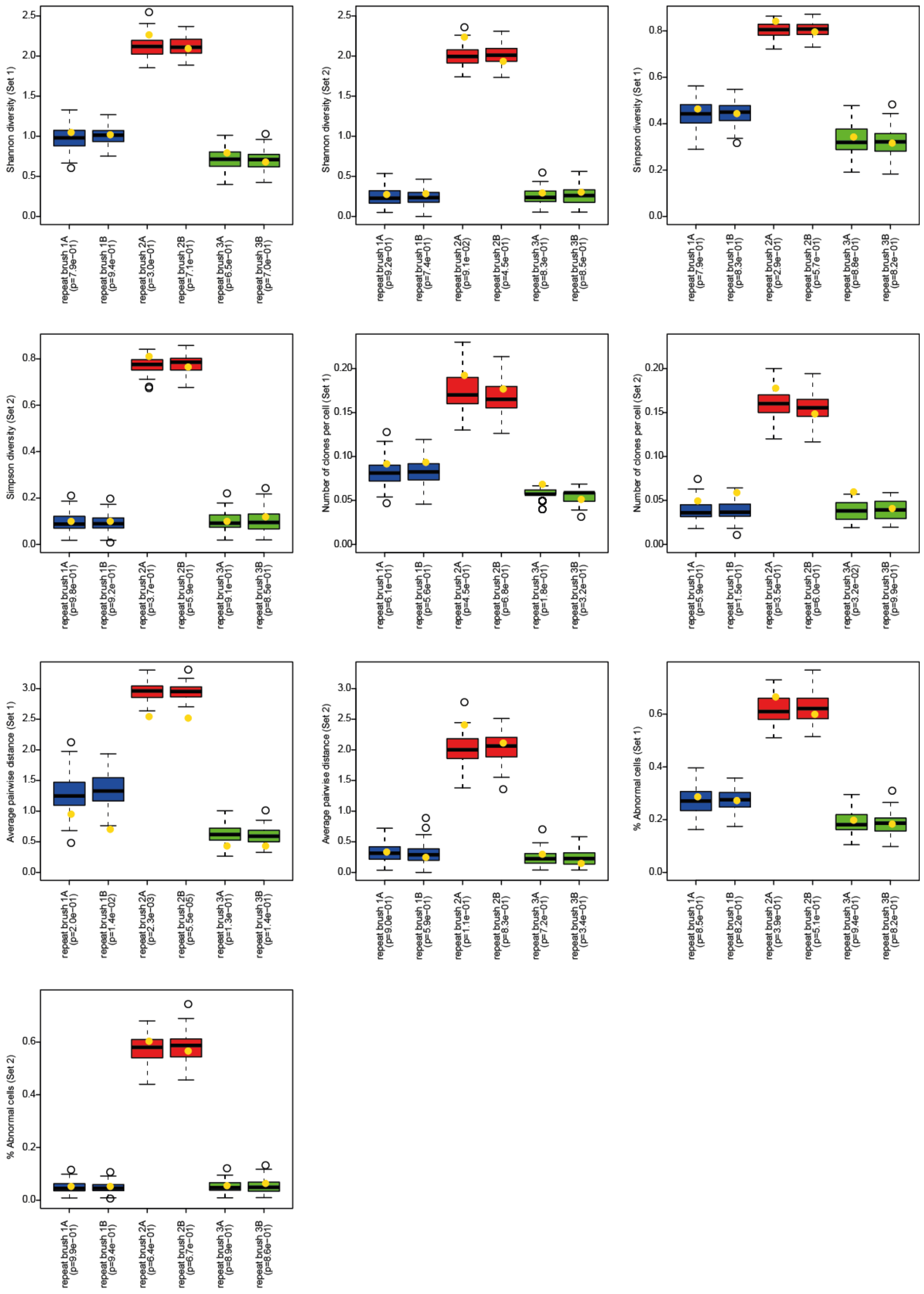

Supplementary Figure 4: Repeat brushes and expected values from resampling simulations. Clones from both repeat brushes were pooled to calculate the 'best' measures of clone size. This distribution was then subjected to 100 resampling experiments for each repeat brush, where in each resampling a draw of as many clones as originally scored by FISH was taken at random, with replacement. The subsequent distributions of each diversity measure were used to compute Z scores for each observation and calculate the relative p-value. Boxes indicate the lower and upper boundaries of the 2nd and 3rd quartiles, whiskers indicate 95% confidence intervals, white dots indicate outliers. Orange dots indicate the empirically measured diversity value in each brush.

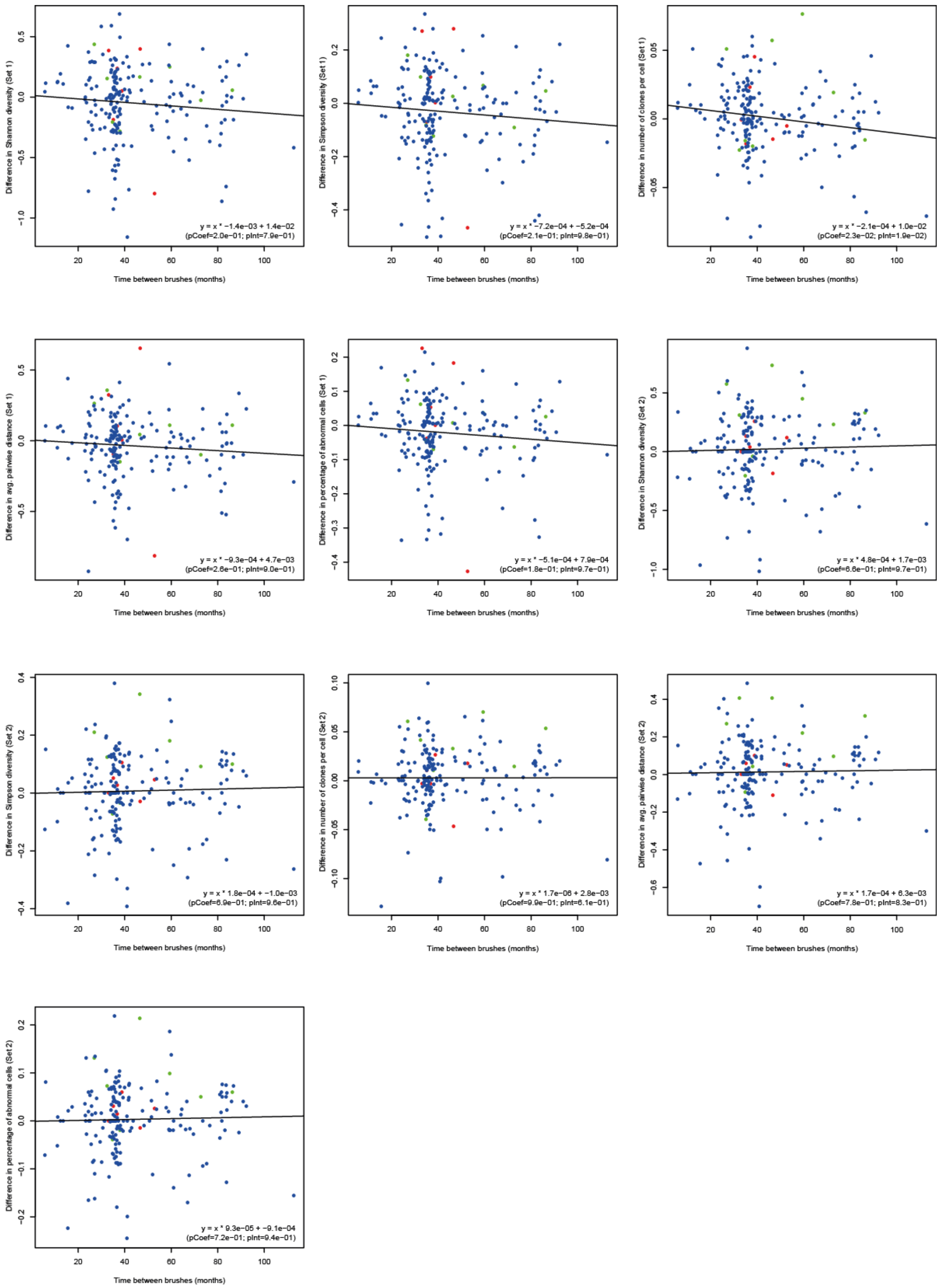

Supplementary Figure 5: Linear fits for differences in all diversity-based measures and time between brushes. Blue dots: non-progressors; red dots: patients progressing after 2nd time point; green dots: patients progressing before 2nd time point.

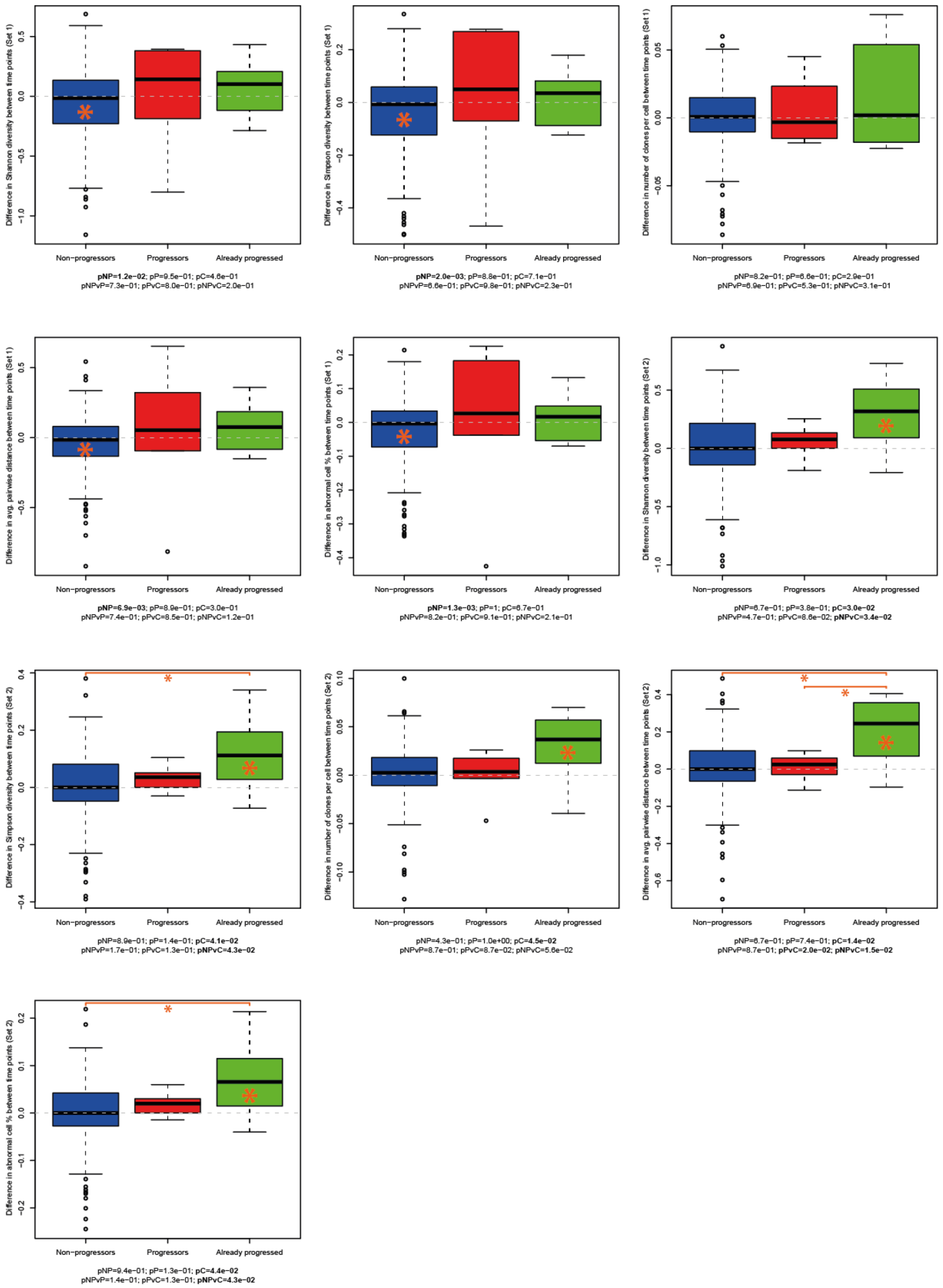

Supplementary Figure 6: Differences in all diversity-based measures between brushes for all three categories of patients. Non-progressors (blue, n=181), Progressors (red, n=6) and Cancers (n=9). One progressor had both a pre-progression brush, included in the progressor set, and a post-progression brush, included in the cancer set. Boxes indicate the lower and upper boundaries of the 2nd and 3rd quartiles, whiskers indicate 95% confidence intervals, white dots indicate outliers. Orange stars indicate statistical significance that the difference between time points is significant (paired t-test, stars in boxes) or that the difference between time points is statistically different between different categories (Kolmogorov-Smirnov test, stars below orange horizontal bars). The p-values are reported below each plot, the first line corresponding to paired t-test between time points while the second line corresponds to KS tests between categories (NP=Non-progressors; P=Progressors; C=Cancers).

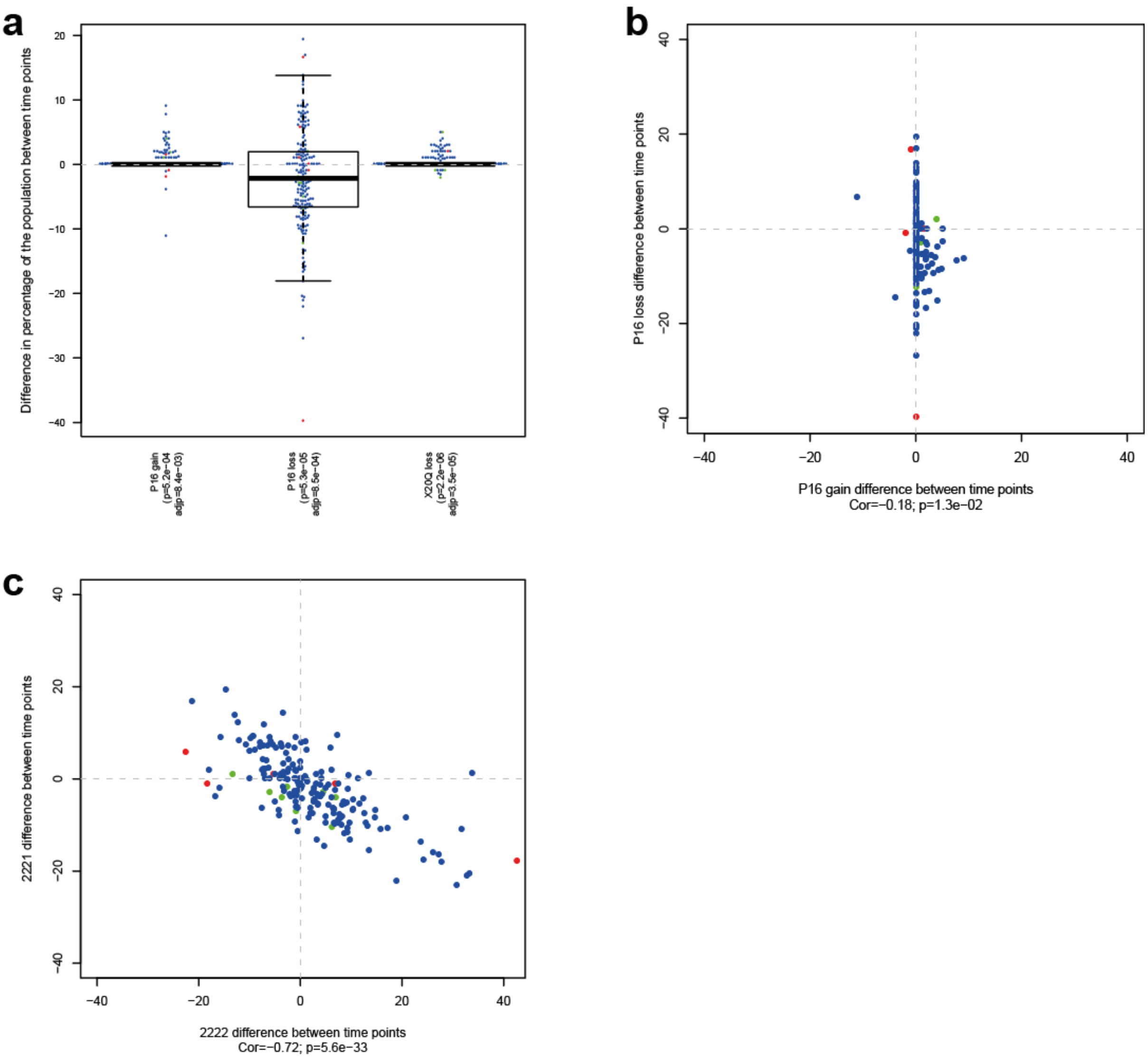

Supplementary Figure 7: p16 evolution between time points. a) Allelic anomalies that are significantly enriched or depleted at the second time point. Boxes indicate the lower and upper boundaries of the 2nd and 3rd quartiles, whiskers indicate 95% confidence intervals. P-values obtained via paired t-tests, adjusted p-values ("adjp") obtained via Bonferroni correction. b) Relation between p16 loss and p16 gain in the population in between time points. c) Relation between the frequencies of the "2221" genotype (p16 loss) and the "2222" genotype (normal) in the population between time points. Blue dots: non-progressors; red dots: patients progressing after 2nd time point; green dots: patients progressing before 2nd time point.

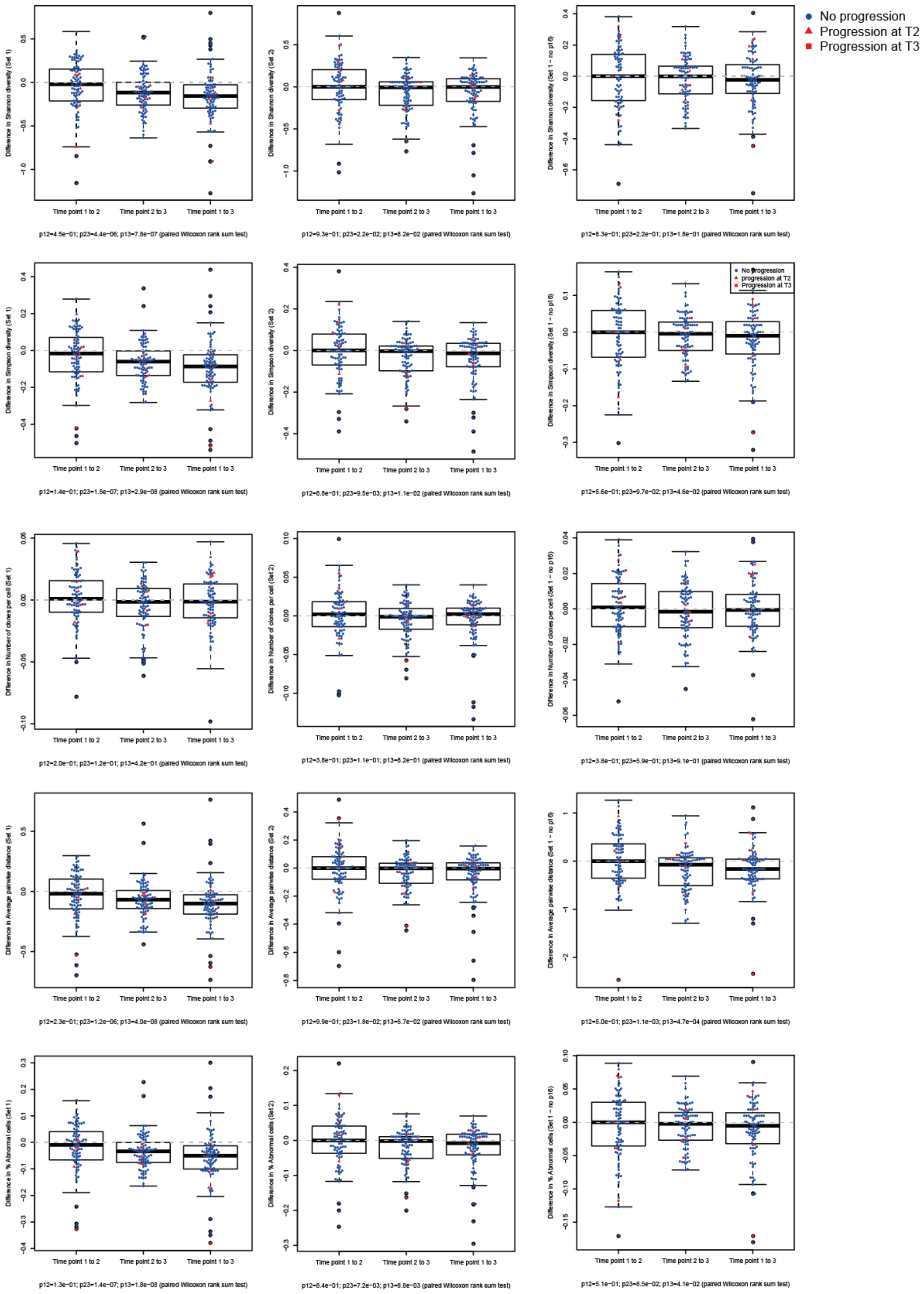

Supplementary Figure 8: Differences in all diversity-based measures between brushes for patients with a third time point brush. Differences observed using Set 1 probes on the left column, using Set 2 probes on the middle column and using Set 1 without the p16 probe on the right column. Boxes indicate the lower and upper boundaries of the 2nd and 3rd quartiles, whiskers indicate 95% confidence intervals, white dots indicate outliers. p12, p23 and p13 respectively stand for the p-values from paired t-tests between the first and second time points, the second and third, and the first and third respectively. P-values were not corrected for multiple testing.

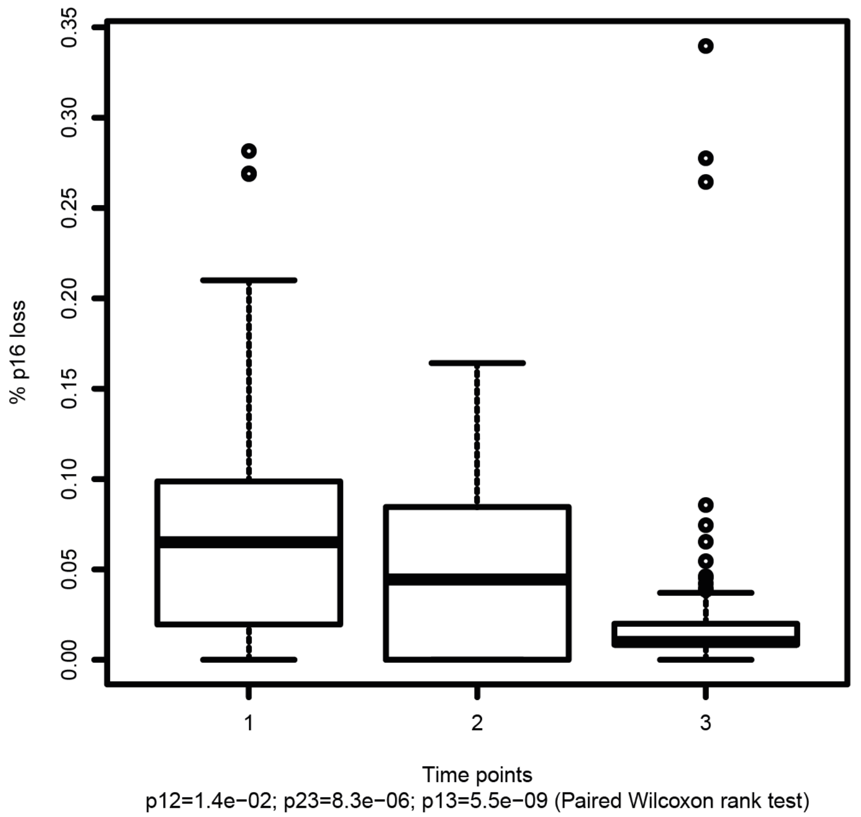

Supplementary Figure 9: Proportion of cells displaying p16 loss at each time point. Only patients with 3 time points available were analysed. Boxes indicate the lower and upper boundaries of the 2nd and 3rd quartiles, whiskers indicate 95% confidence intervals, white dots indicate outliers. p12, p23 and p13 respectively stand for the p-values from paired Wilcoxon rank tests between the first and second time points, the second and third, the first and third. P-values were not corrected for multiple testing.

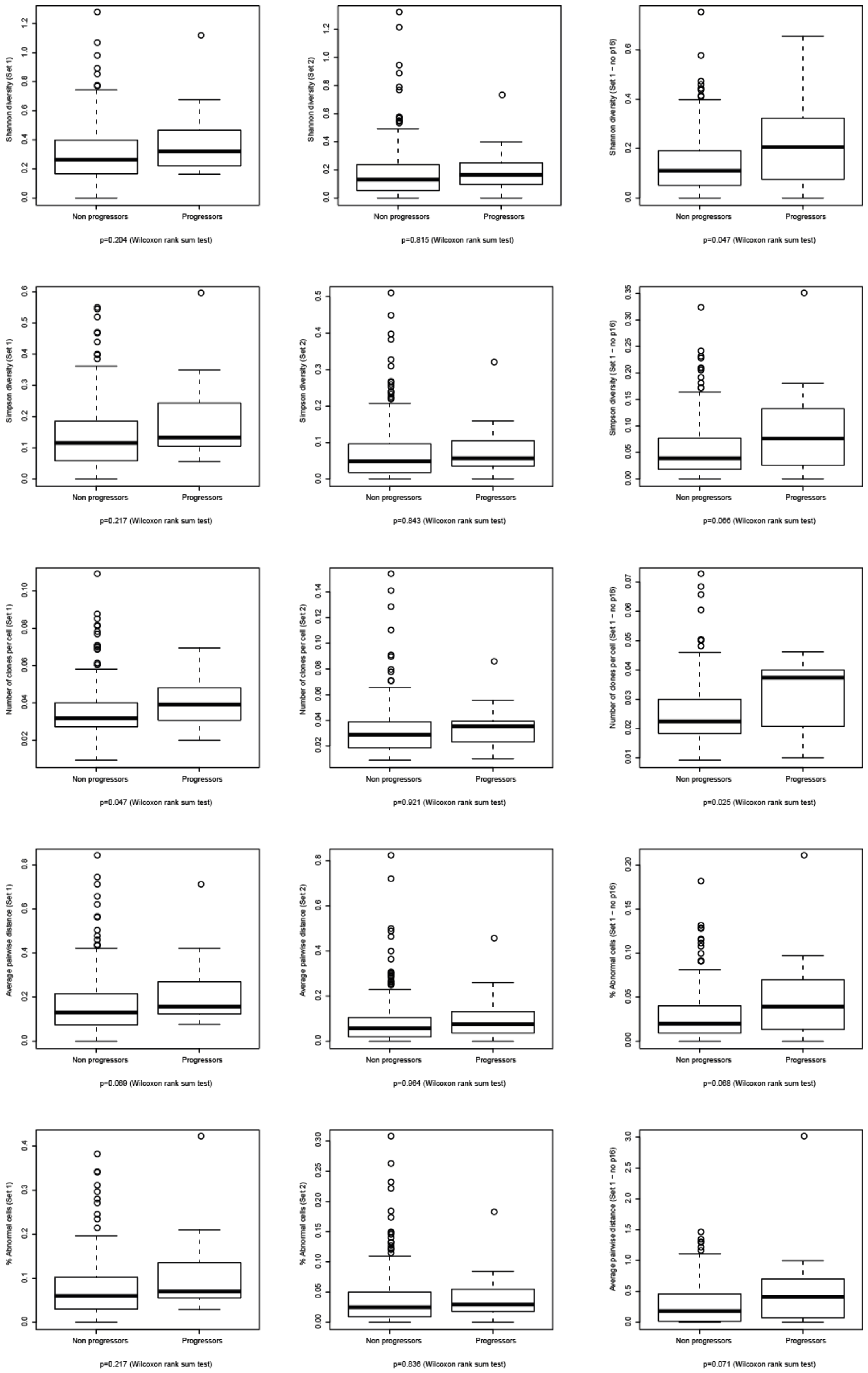

Supplementary Figure 10: Differences in diversity between progressor and non-progressor diversity at the third time point. Boxes indicate the lower and upper boundaries of the 2nd and 3rd quartiles, whiskers indicate 95% confidence intervals, white dots indicate outliers. P-values were not corrected for multiple testing.

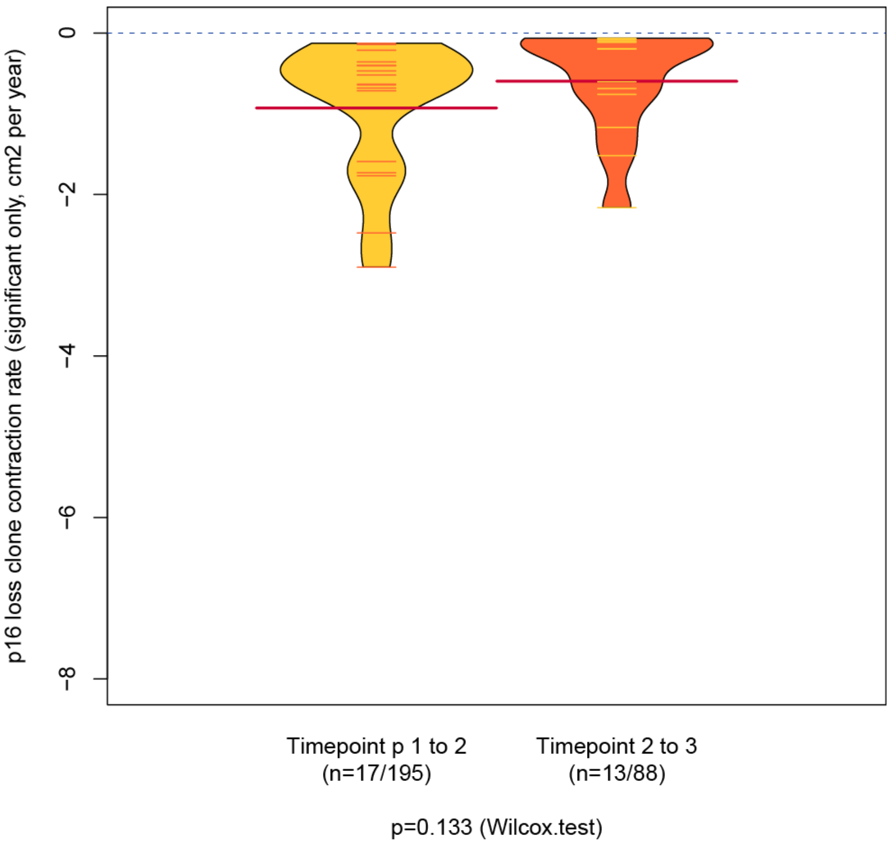

Supplementary Figure 11: Growth rates linked to contraction of p16 loss clones. Growth rates for significant contraction occurring between the first and second time points (left) and between the second and third time points (right). Dashes are individual measurements; red lines annotated with a number represent the mean.

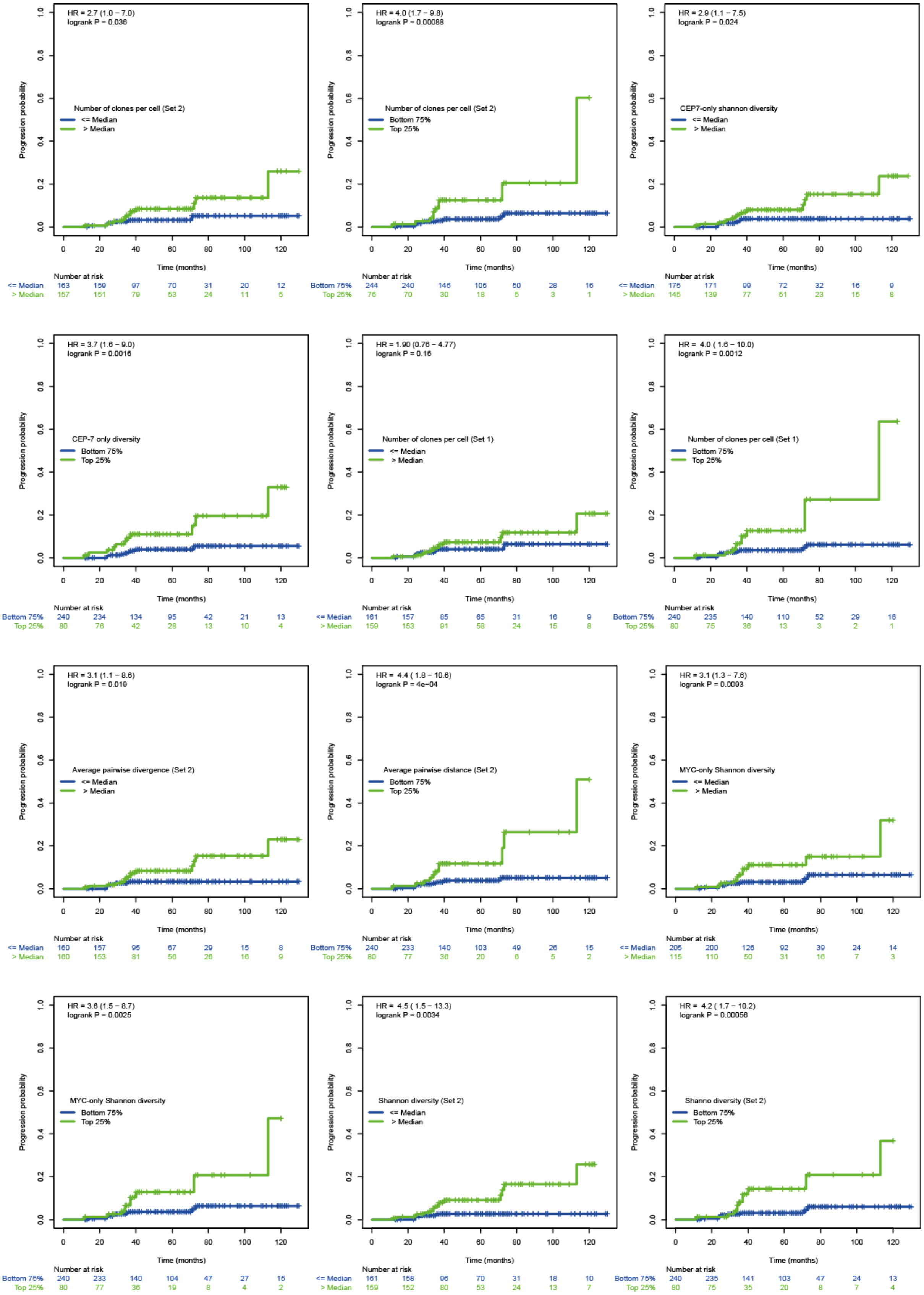
HR = 2.7 (1.0 − 7.0)
logrank P = 0.036
Number of clones per cell (Set 2)
<= Median
> Median
Progression probability
Time (months)
Number at risk
<= Median 163 159 97 70 31 20 12
> Median 157 151 79 53 24 11 5
HR = 4.0 (1.7 − 9.8)
logrank P = 0.00088
Number of clones per cell (Set 2)
Bottom 75%
Top 25%
Progression probability
Time (months)
Number at risk
Bottom 75% 244 240 146 105 50 28 16
Top 25% 76 70 30 18 5 3 1
HR = 2.9 (1.1 − 7.5)
logrank P = 0.024
CEP7-only shannon diversity
<= Median
> Median
Progression probability
Time (months)
Number at risk
<= Median 175 171 99 72 32 16 9
> Median 145 139 77 51 23 15 8
HR = 3.7 (1.6 − 9.0)
logrank P = 0.0016
CEP-7 only diversity
Bottom 75%
Top 25%
Progression probability
Time (months)
Number at risk
Bottom 75% 240 234 134 95 42 21 13
Top 25% 80 76 42 28 13 10 4
HR = 1.90 (0.76 − 4.77)
logrank P = 0.16
Number of clones per cell (Set 1)
<= Median
> Median
Progression probability
Time (months)
Number at risk
<= Median 161 157 85 65 31 16 9
> Median 159 153 91 58 24 15 8
HR = 4.0 (1.6 − 10.0)
logrank P = 0.0012
Number of clones per cell (Set 1)
Bottom 75%
Top 25%
Progression probability
Time (months)
Number at risk
Bottom 75% 240 235 140 110 52 29 16
Top 25% 80 75 36 13 3 2 1
HR = 3.1 (1.1 − 8.6)
logrank P = 0.019
Average pairwise divergence (Set 2)
<= Median
> Median
Progression probability
Time (months)
Number at risk
<= Median 160 157 95 67 29 15 8
> Median 160 153 81 56 26 16 9
HR = 4.4 (1.8 − 10.6)
logrank P = 4e−04
Average pairwise distance (Set 2)
Bottom 75%
Top 25%
Progression probability
Time (months)
Number at risk
Bottom 75% 240 233 140 103 49 26 15
Top 25% 80 77 36 20 6 5 2
HR = 3.1 (1.3 − 7.6)
logrank P = 0.0093
MYC-only Shannon diversity
<= Median
> Median
Progression probability
Time (months)
Number at risk
<= Median 205 200 126 92 39 24 14
> Median 115 110 50 31 16 7 3
HR = 3.6 (1.5 − 8.7)
logrank P = 0.0025
MYC-only Shannon diversity
Bottom 75%
Top 25%
Progression probability
Time (months)
Number at risk
Bottom 75% 240 233 140 104 47 27 15
Top 25% 80 77 36 19 8 4 2
HR = 4.5 (1.5 − 13.3)
logrank P = 0.0034
Shannon diversity (Set 2)
<= Median
> Median
Progression probability
Time (months)
Number at risk
<= Median 161 158 96 70 31 18 10
> Median 159 152 80 53 24 13 7
HR = 4.2 (1.7 − 10.2)
logrank P = 0.00056
Shanno diversity (Set 2)
Bottom 75%
Top 25%
Progression probability
Time (months)
Number at risk
Bottom 75% 240 235 141 103 47 24 13
Top 25% 80 75 35 20 8 7 4

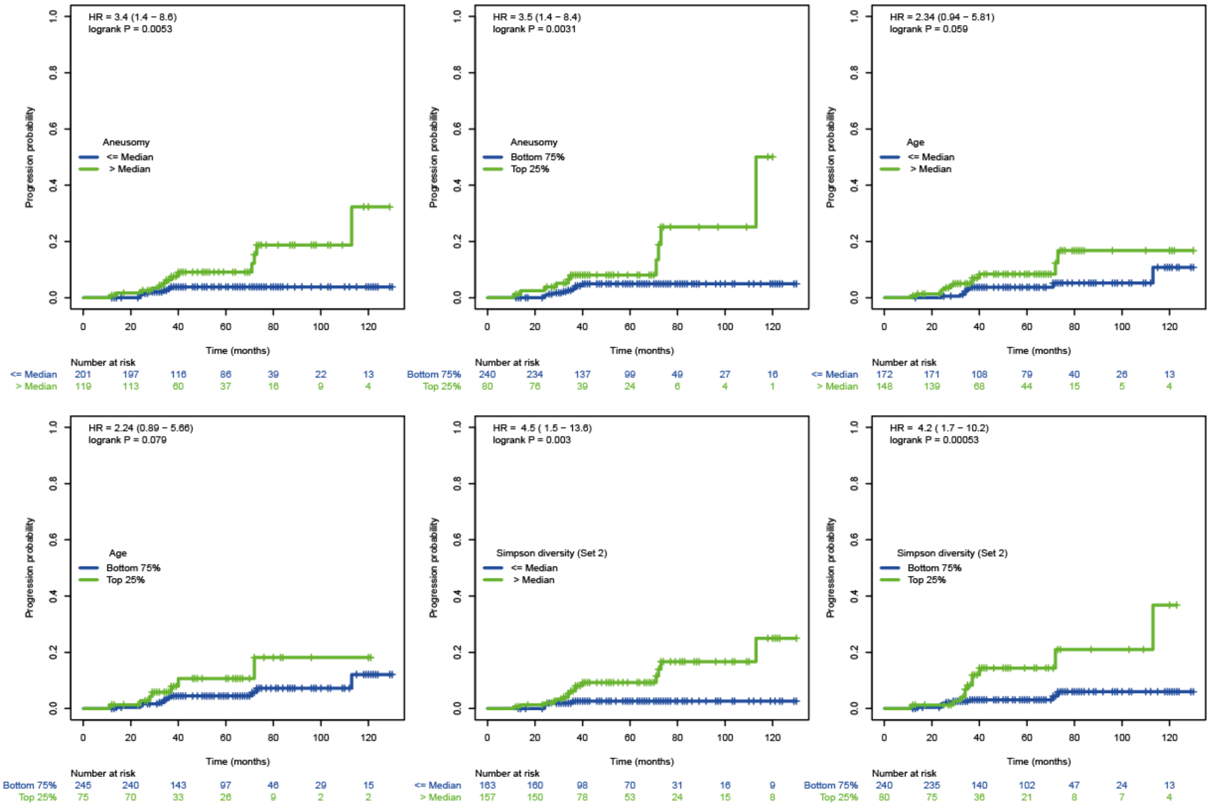

Supplementary Figure 12: Kaplan-Meier curves for patient stratification. Colored lines indicate the proportion of patients progressing to cancer, vertical bars indicate right-censoring (end of follow-up data).

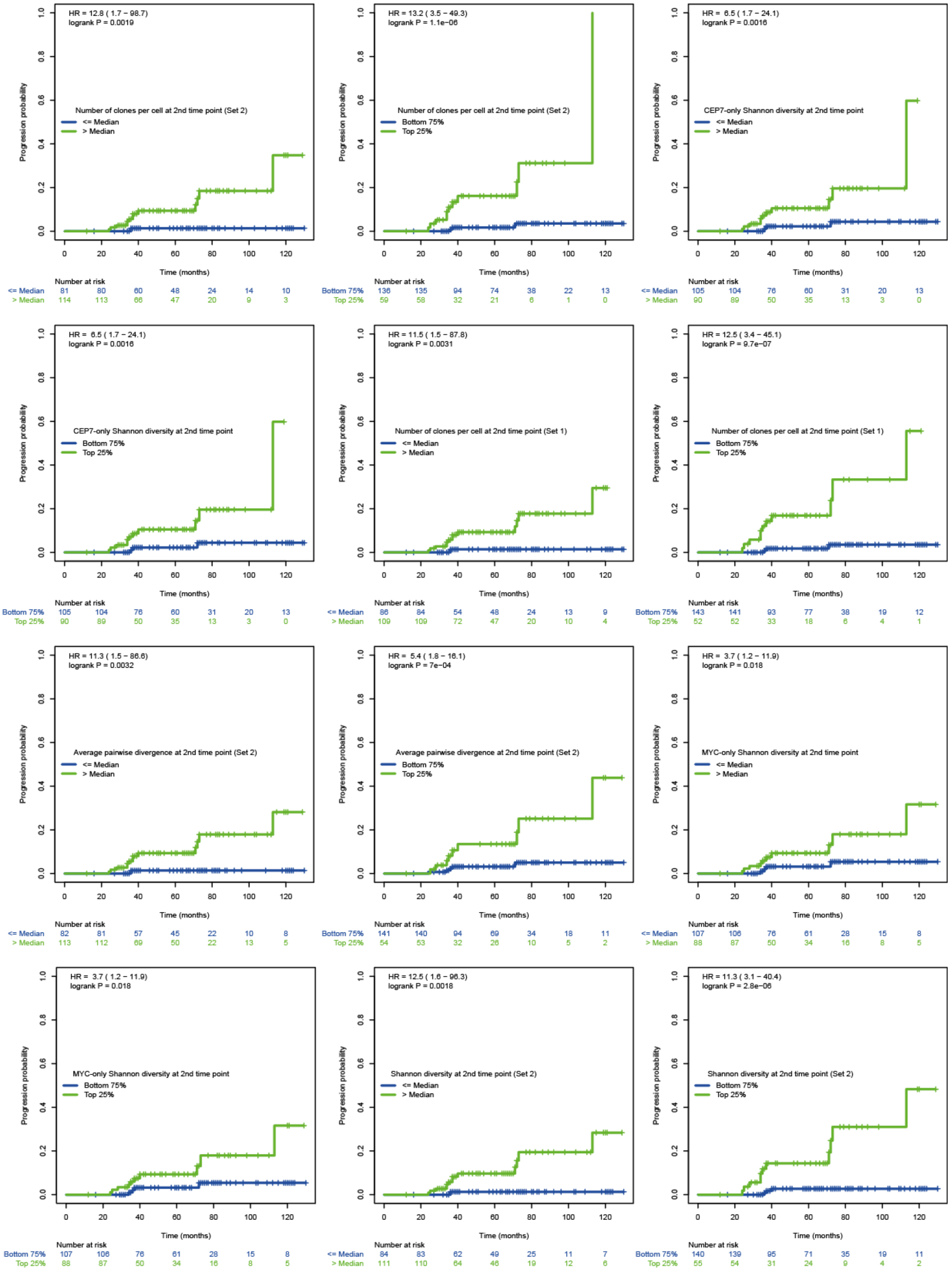

HR = 12.8 (1.7 − 98.7)
logrank P = 0.0019
Number of clones per cell at 2nd time point (Set 2)
<= Median
> Median
Progression probability
Time (months)
Number at risk
<= Median 81 80 60 48 24 14 10
> Median 114 113 66 47 20 9 3
HR = 13.2 (3.5 − 49.3)
logrank P = 1.1e−06
Number of clones per cell at 2nd time point (Set 2)
Bottom 75%
Top 25%
Number at risk
Bottom 75% 136 135 94 74 38 22 13
Top 25% 59 58 32 21 6 1 0
HR = 6.5 (1.7 − 24.1)
logrank P = 0.0016
CEP7-only Shannon diversity at 2nd time point
<= Median
> Median
Number at risk
<= Median 105 104 76 60 31 20 13
> Median 90 89 50 35 13 3 0
HR = 6.5 (1.7 − 24.1)
logrank P = 0.0016
CEP7-only Shannon diversity at 2nd time point
Bottom 75%
Top 25%
Number at risk
Bottom 75% 105 104 76 60 31 20 13
Top 25% 90 89 50 35 13 3 0
HR = 11.5 (1.5 − 87.8)
logrank P = 0.0031
Number of clones per cell at 2nd time point (Set 1)
<= Median
> Median
Number at risk
<= Median 86 84 54 48 24 13 9
> Median 109 109 72 47 20 10 4
HR = 12.5 (3.4 − 45.1)
logrank P = 9.7e−07
Number of clones per cell at 2nd time point (Set 1)
Bottom 75%
Top 25%
Number at risk
Bottom 75% 143 141 93 77 38 19 12
Top 25% 52 52 33 18 6 4 1
HR = 11.3 (1.5 − 86.6)
logrank P = 0.0032
Average pairwise divergence at 2nd time point (Set 2)
<= Median
> Median
Number at risk
<= Median 82 81 57 45 22 10 8
> Median 113 112 69 50 22 13 5
HR = 5.4 (1.8 − 16.1)
logrank P = 7e−04
Average pairwise divergence at 2nd time point (Set 2)
Bottom 75%
Top 25%
Number at risk
Bottom 75% 141 140 94 69 34 18 11
Top 25% 54 53 32 26 10 5 2
HR = 3.7 (1.2 − 11.9)
logrank P = 0.018
MYC-only Shannon diversity at 2nd time point
<= Median
> Median
Number at risk
<= Median 107 106 76 61 28 15 8
> Median 88 87 50 34 16 8 5
HR = 3.7 (1.2 − 11.9)
logrank P = 0.018
MYC-only Shannon diversity at 2nd time point
Bottom 75%
Top 25%
Number at risk
Bottom 75% 107 106 76 61 28 15 8
Top 25% 88 87 50 34 16 8 5
HR = 12.5 (1.6 − 96.3)
logrank P = 0.0018
Shannon diversity at 2nd time point (Set 2)
<= Median
> Median
Number at risk
<= Median 84 83 62 49 25 11 7
> Median 111 110 64 46 19 12 6
HR = 11.3 (3.1 − 40.4)
logrank P = 2.8e−06
Shannon diversity at 2nd time point (Set 2)
Bottom 75%
Top 25%
Number at risk
Bottom 75% 140 139 95 71 35 19 11
Top 25% 55 54 31 24 9 4 2

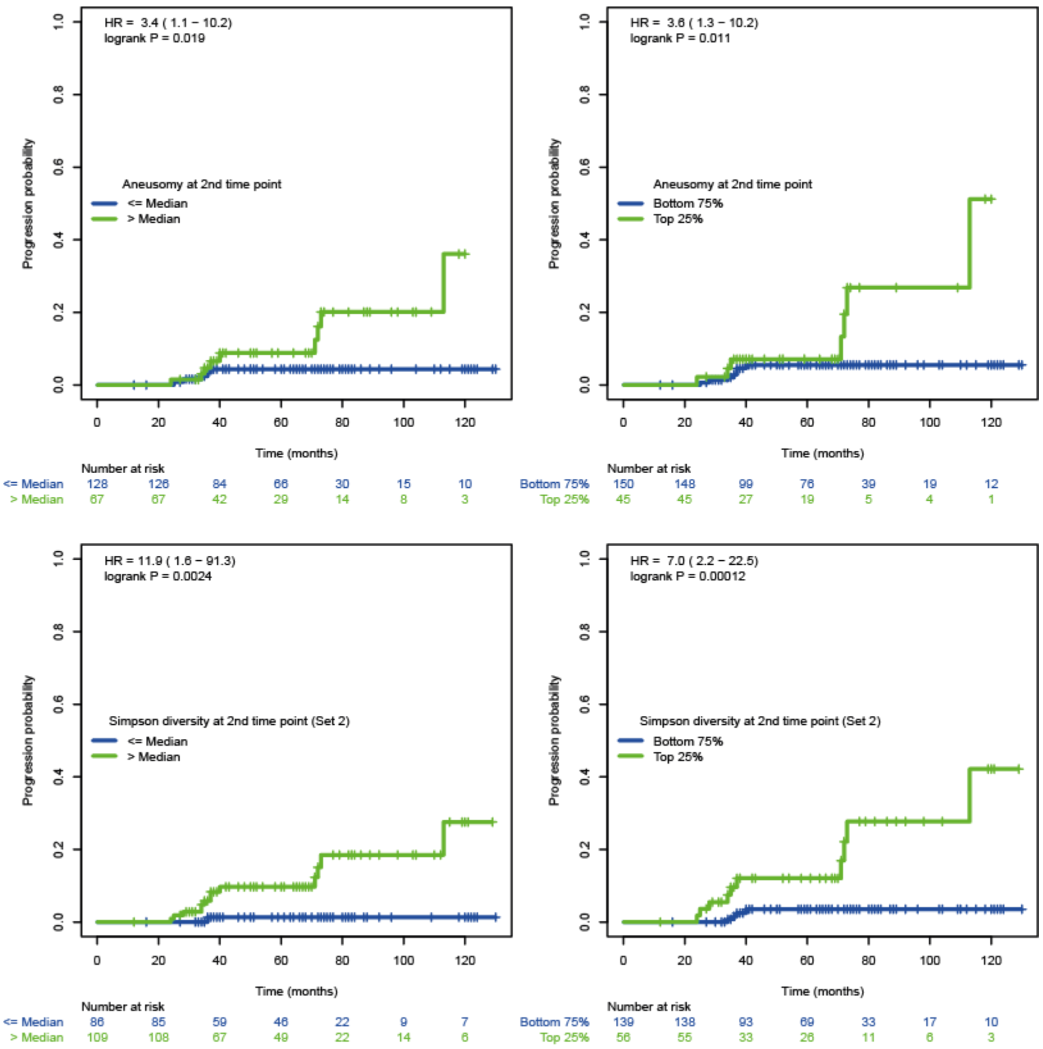

Supplementary Figure 13: Kaplan-Meier curves for patient stratification using 2nd time point values. Colored lines indicate the proportion of patients progressing to cancer, vertical bars indicate right-censoring (end of follow-up data). Thresholds are based on the 1st time point distributions of each stratifying variable.

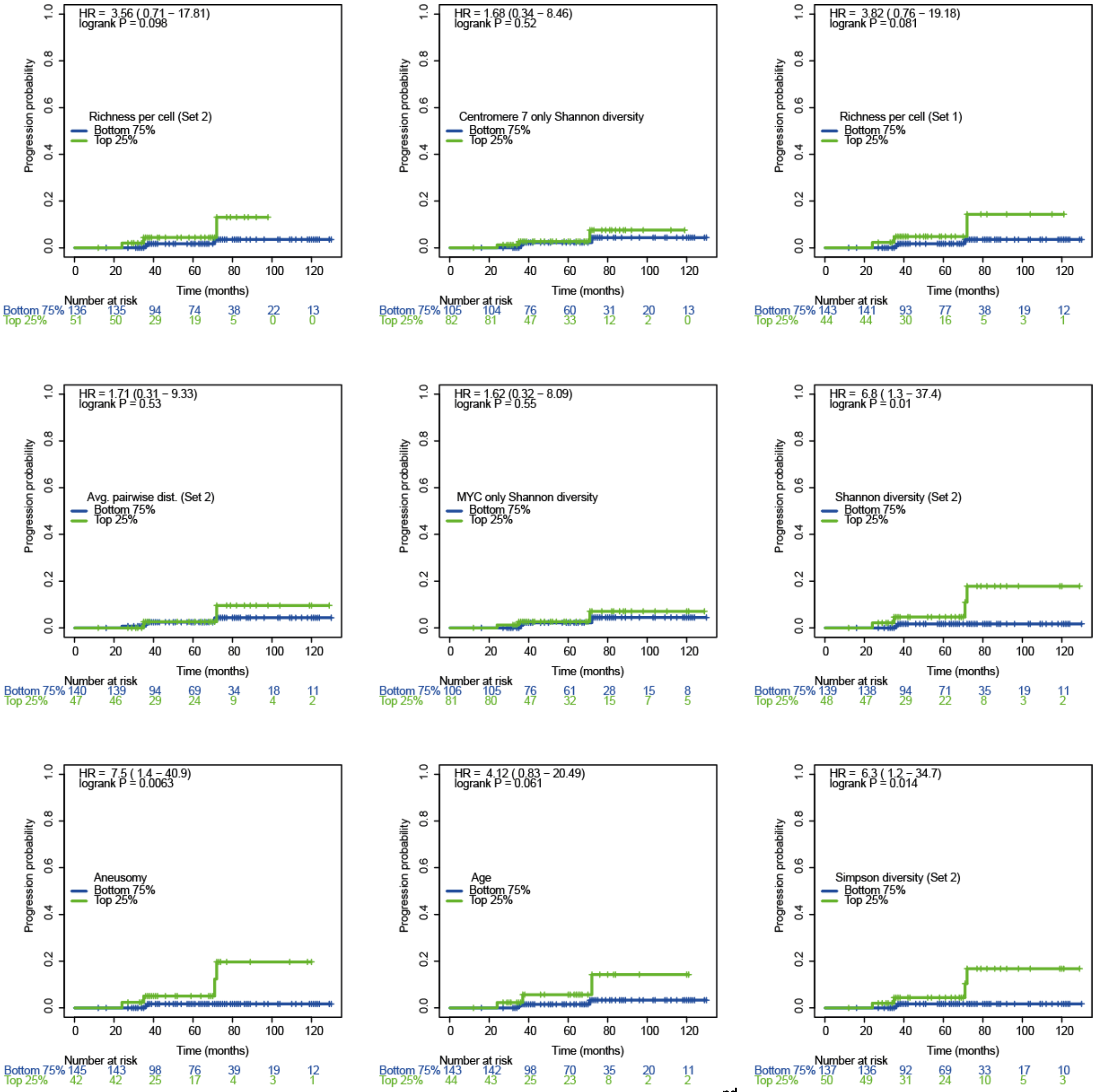

Supplementary Figure 14: Kaplan-Meier curves for patient stratification using 2nd time point values removing patients having already progressed to cancer. Colored lines indicate the proportion of patients progressing to cancer, vertical bars indicate right-censoring (end of follow-up data). Thresholds are based on the 1st time point distributions of each stratifying variable.

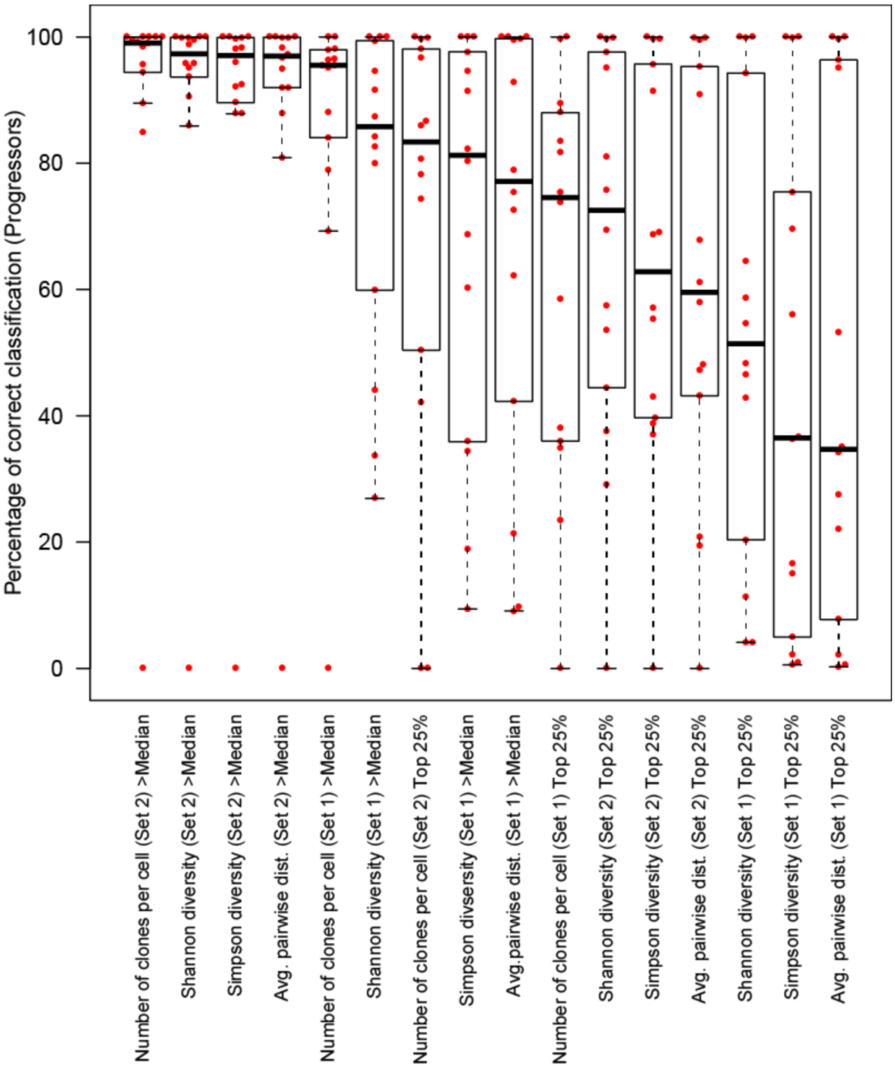

Supplementary Figure 15: Bootstrapped diversity-based classification of progressors. Percentage of all simulations each progressor is correctly identified. Boxes indicate the lower and upper boundaries of the 2nd and 3rd quartiles, whiskers indicate 95% confidence intervals, white dots indicate outliers. For each sample with n1 and n2 cells scored at the 1st and 2nd time point, respectively, we generated 1,000 draws of n1 cells and 1,000 draws of n2 cells from the pooled distribution of n1+n2 cells. Red dots represent the percentage of the 2,000 total draws in which each progressor was identified as high-risk using the thresholds defined using 1st time point diversity measures (> median or upper quartile as high risk).

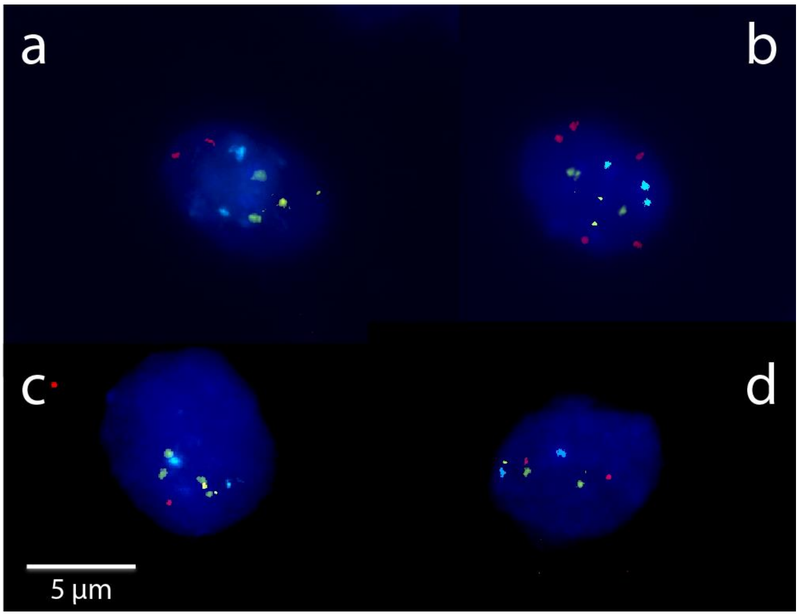

Supplementary Figure 16: Multicolour FISH. Representative examples of FISH signal patterns seen in Barrett's esophagus with Set 1 comprising CEP17 [aqua], Her-2/neu [green], p53 [gold], and p16 (9p21) [red] and set 2 comprising CEP7 [aqua], CEP17 [green], 20q [gold], and MYC [red]. a) set 2: normal cell (2 signals of each probe). b) set 2: multiple genetic abnormalities including MYC gain (>2 red signals) and aneusomy for CEP 7 (>2 aqua signals). c) set 1: homozygous p16 loss (1 red signal) and aneusomy for CEP 17 (>2 green signals). d) set 2: homozygous p53 loss (1 gold signal).

SUPPLEMENTARY TABLES

Supplementary Table 1: Sample with significant differences in diversity compared to expectations from sampling bias. Z = Z-score of observed change in diversity compared to resampled distribution; p= p-value; Corrected p = Bonferroni corrected p-value. Red indicates a diversity increase, yellow a decrease, green highlights significance.

		Shannon Diversity (Set 1)			Shannon Diversity (Set 2)			Simpson Diversity (Set 1)			Simpson Diversity (Set 2)			Nb. of clones per cell (Set 1)			Nb. of clones per cell (Set 2)			Avg. pairwise dist. (Set 1)			Avg. pairwise dist. (Set 2)		
Patient	Progress	*Z*	*p*	*Corrected p*	*Z*	*p*	*Corrected p*	*Z*	*p*	*Corrected p*	*Z*	*p*	*Corrected p*	*Z*	*p*	*Corrected p*	*Z*	*p*	*Corrected p*	*Z*	*p*	*Corrected p*	*Z*	*p*	*Corrected p*
4	Yes	1.14	2.6E-01	1.0E+00	3.73	1.9E-04	3.7E-02	0.27	7.9E-01	1.0E+00	3.61	3.1E-04	6.0E-02	2.41	1.6E-02	1.0E+00	3.58	3.4E-04	6.6E-02	0.40	6.9E-01	1.0E+00	2.56	1.0E-02	1.0E+00
79	Yes	-6.37	1.9E-10	3.8E-08	0.98	3.3E-01	1.0E+00	-6.60	4.0E-11	7.9E-09	0.75	4.5E-01	1.0E+00	-1.23	2.2E-01	1.0E+00	1.84	6.5E-02	1.0E+00	-6.20	5.5E-10	1.1E-07	0.76	4.5E-01	1.0E+00
110	Yes	2.54	1.1E-02	1.0E+00	-0.92	3.6E-01	1.0E+00	3.54	3.9E-04	7.7E-02	-0.38	7.0E-01	1.0E+00	-1.32	1.9E-01	1.0E+00	-2.55	1.1E-02	1.0E+00	4.03	5.5E-05	1.1E-02	-0.91	3.6E-01	1.0E+00
2	No	-4.42	9.6E-06	1.9E-03	2.35	1.9E-02	1.0E+00	-4.87	1.1E-06	2.2E-04	2.35	1.9E-02	1.0E+00	-1.89	5.8E-02	1.0E+00	1.74	8.2E-02	1.0E+00	-4.58	4.7E-06	9.1E-04	2.35	1.9E-02	1.0E+00
17	No	0.45	6.6E-01	1.0E+00	-3.96	7.6E-05	1.5E-02	-0.62	5.3E-01	1.0E+00	-3.83	1.3E-04	2.4E-02	2.86	4.3E-03	8.3E-01	-3.17	1.5E-03	3.0E-01	-0.07	9.4E-01	1.0E+00	-3.80	1.4E-04	2.8E-02
21	No	-5.38	7.6E-08	1.5E-05	-3.02	2.5E-03	4.9E-01	-6.13	8.8E-10	1.7E-07	-3.10	1.9E-03	3.7E-01	-2.08	3.8E-02	1.0E+00	NA	NA	NA	-6.47	9.7E-11	1.9E-08	-4.05	5.2E-05	1.0E-02
27	No	-4.90	9.7E-07	1.9E-04	NA	NA	NA	-4.73	2.2E-06	4.4E-04	NA	NA	NA	-3.12	1.8E-03	3.5E-01	NA	NA	NA	-4.64	3.5E-06	6.9E-04	NA	NA	NA
33	No	-4.99	6.2E-07	1.2E-04	1.82	6.8E-02	1.0E+00	-4.76	1.9E-06	3.8E-04	1.69	9.2E-02	1.0E+00	-4.61	4.0E-06	7.8E-04	2.02	4.3E-02	1.0E+00	-4.84	1.3E-06	2.6E-04	1.69	9.2E-02	1.0E+00
37	No	-5.31	1.1E-07	2.2E-05	NA	NA	NA	-5.08	3.9E-07	7.5E-05	NA	NA	NA	-3.74	1.9E-04	3.6E-02	NA	NA	NA	-4.97	6.6E-07	1.3E-04	NA	NA	NA
61	No	-3.06	2.2E-03	4.4E-01	NA	NA	NA	-2.89	3.8E-03	7.4E-01	NA	NA	NA	-3.76	1.7E-04	3.4E-02	NA	NA	NA	-2.89	3.8E-03	7.4E-01	NA	NA	NA
73	No	-5.12	3.1E-07	6.1E-05	-2.71	6.8E-03	1.0E+00	-4.83	1.4E-06	2.7E-04	-2.60	9.2E-03	1.0E+00	-3.43	6.1E-04	1.2E-01	-2.26	2.4E-02	1.0E+00	-4.79	1.7E-06	3.2E-04	-2.61	9.1E-03	1.0E+00
98	No	2.68	7.3E-03	1.0E+00	-1.71	8.7E-02	1.0E+00	2.46	1.4E-02	1.0E+00	-1.64	1.0E-01	1.0E+00	4.08	4.4E-05	8.6E-03	-1.58	1.2E-01	1.0E+00	2.46	1.4E-02	1.0E+00	-1.64	1.0E-01	1.0E+00
112	No	-0.24	8.1E-01	1.0E+00	-4.56	5.1E-06	9.9E-04	-0.73	4.6E-01	1.0E+00	-4.34	1.4E-05	2.7E-03	1.33	1.8E-01	1.0E+00	-3.72	2.0E-04	3.8E-02	-0.83	4.1E-01	1.0E+00	-4.13	3.6E-05	7.0E-03
117	No	-3.99	6.5E-05	1.3E-02	NA	NA	NA	-3.90	9.7E-05	1.9E-02	NA	NA	NA	-2.96	3.0E-03	5.9E-01	NA	NA	NA	-3.91	9.1E-05	1.8E-02	NA	NA	NA
130	No	-6.35	2.2E-10	4.2E-08	NA	NA	NA	-6.08	1.2E-09	2.3E-07	NA	NA	NA	-5.13	2.9E-07	5.7E-05	NA	NA	NA	-5.23	1.7E-07	3.3E-05	NA	NA	NA
133	No	1.21	2.3E-01	1.0E+00	5.15	2.6E-07	5.1E-05	0.90	3.7E-01	1.0E+00	4.91	9.2E-07	1.8E-04	1.93	5.3E-02	1.0E+00	4.31	1.7E-05	3.2E-03	1.51	1.3E-01	1.0E+00	4.62	3.8E-06	7.5E-04
185	No	1.52	1.3E-01	1.0E+00	3.19	1.4E-03	2.7E-01	1.31	1.9E-01	1.0E+00	2.93	3.3E-03	6.5E-01	2.70	6.9E-03	1.0E+00	3.70	2.1E-04	4.2E-02	1.31	1.9E-01	1.0E+00	2.04	4.1E-02	1.0E+00
194	No	4.02	5.8E-05	1.1E-02	0.76	4.5E-01	1.0E+00	3.81	1.4E-04	2.7E-02	0.69	4.9E-01	1.0E+00	3.57	3.5E-04	6.9E-02	0.98	3.3E-01	1.0E+00	3.80	1.4E-04	2.8E-02	0.69	4.9E-01	1.0E+00
200	No	1.72	8.6E-02	1.0E+00	3.30	9.6E-04	1.9E-01	1.52	1.3E-01	1.0E+00	3.03	2.4E-03	4.8E-01	1.95	5.1E-02	1.0E+00	3.85	1.2E-04	2.3E-02	1.59	1.1E-01	1.0E+00	2.91	3.6E-03	7.1E-01
218	No	-6.80	1.0E-11	2.0E-09	NA	NA	NA	-6.33	2.5E-10	4.8E-08	NA	NA	NA	-6.18	6.6E-10	1.3E-07	NA	NA	NA	-6.15	7.7E-10	1.5E-07	NA	NA	NA
226	No	-2.43	1.5E-02	1.0E+00	0.81	4.2E-01	1.0E+00	-2.12	3.4E-02	1.0E+00	0.61	5.4E-01	1.0E+00	-15.78	4.3E-56	8.4E-54	1.44	1.5E-01	1.0E+00	-2.78	5.4E-03	1.0E+00	0.90	3.7E-01	1.0E+00
229	No	0.00	1.0E+00	1.0E+00	4.57	4.9E-06	9.6E-04	0.65	5.1E-01	1.0E+00	4.44	9.2E-06	1.8E-03	-1.84	6.5E-02	1.0E+00	3.57	3.6E-04	6.9E-02	-0.13	8.9E-01	1.0E+00	4.34	1.4E-05	2.8E-03
230	No	-2.45	1.4E-02	1.0E+00	-3.75	1.8E-04	3.5E-02	-1.40	1.6E-01	1.0E+00	-3.42	6.2E-04	1.2E-01	-3.76	1.7E-04	3.4E-02	-3.91	9.4E-05	1.8E-02	-2.65	8.1E-03	1.0E+00	-3.37	7.5E-04	1.5E-01
235	No	-3.13	1.7E-03	3.4E-01	NA	NA	NA	-2.89	3.9E-03	7.6E-01	NA	NA	NA	-3.99	6.6E-05	1.3E-02	NA	NA	NA	-2.72	6.6E-03	1.0E+00	NA	NA	NA
244	No	4.09	4.2E-05	8.3E-03	2.53	1.1E-02	1.0E+00	3.43	6.1E-04	1.2E-01	2.15	3.1E-02	1.0E+00	3.86	1.1E-04	2.2E-02	2.99	2.8E-03	5.4E-01	3.59	3.2E-04	6.3E-02	2.21	2.7E-02	1.0E+00
248	No	-6.02	1.8E-09	3.5E-07	-4.31	1.6E-05	3.2E-03	-5.85	4.9E-09	9.6E-07	-3.94	8.0E-05	1.6E-02	-4.16	3.1E-05	6.1E-03	-4.42	1.0E-05	2.0E-03	-5.58	2.4E-08	4.7E-06	-4.15	3.3E-05	6.5E-03
259	No	2.57	1.0E-02	1.0E+00	-1.58	1.1E-01	1.0E+00	3.00	2.7E-03	5.3E-01	-1.44	1.5E-01	1.0E+00	0.07	9.5E-01	1.0E+00	-2.04	4.1E-02	1.0E+00	3.77	1.6E-04	3.2E-02	-1.43	1.5E-01	1.0E+00
266	No	-2.98	2.8E-03	5.5E-01	-4.22	2.4E-05	4.8E-03	-2.85	4.3E-03	8.4E-01	-4.00	6.4E-05	1.2E-02	-2.04	4.1E-02	1.0E+00	-3.63	2.8E-04	5.4E-02	-2.74	6.1E-03	1.0E+00	-4.00	6.4E-05	1.2E-02
281	No	2.02	4.4E-02	1.0E+00	-4.65	3.3E-06	6.4E-04	2.32	2.0E-02	1.0E+00	-4.46	8.0E-06	1.6E-03	0.91	3.6E-01	1.0E+00	-4.25	2.2E-05	4.2E-03	2.41	1.6E-02	1.0E+00	-3.60	3.2E-04	6.2E-02
286	No	3.68	2.4E-04	4.6E-02	1.91	5.6E-02	1.0E+00	3.96	7.5E-05	1.5E-02	1.83	6.7E-02	1.0E+00	1.07	2.9E-01	1.0E+00	1.67	9.6E-02	1.0E+00	2.80	5.1E-03	9.9E-01	1.83	6.7E-02	1.0E+00
316	No	-2.65	8.1E-03	1.0E+00	-3.75	1.8E-04	3.5E-02	-2.69	7.2E-03	1.0E+00	-3.77	1.7E-04	3.2E-02	-2.07	3.8E-02	1.0E+00	-2.91	3.6E-03	7.0E-01	-2.84	4.5E-03	8.8E-01	-3.83	1.3E-04	2.5E-02
320	No	-4.47	7.8E-06	1.5E-03	-2.02	4.4E-02	1.0E+00	-4.19	2.8E-05	5.5E-03	-1.87	6.2E-02	1.0E+00	-4.09	4.3E-05	8.3E-03	-2.25	2.4E-02	1.0E+00	-3.86	1.1E-04	2.2E-02	-1.86	6.3E-02	1.0E+00

Supplementary Table 2: Significant binomial tests for clonal expansion/contraction detection.

Clone	Patient	Set	t2Obs	t2N	t2Freq	t1Freq	p	Bonferroni p	Growth rate g_i (% population per month)	Growth rate G_i (cm^2 per month)	λ	Description	Time between brushes (months)
2222	4	2	59	81	0.73	0.94	8.0E-10	9.4E-07	-0.0046	-1.6643	-0.004	Normal genotype decrease	46.5
2220	79	1	0	87	0.00	0.22	7.3E-10	8.5E-07	-0.0042	-1.8851	-0.008	p16 loss decrease	52.8
2222	79	1	78	87	0.90	0.47	7.2E-17	8.4E-14	0.0081	3.6549	0.018	Normal genotype increase	52.8
2222	110	1	66	86	0.77	0.95	7.1E-09	8.2E-06	-0.0039	-0.3538	-0.003	Normal genotype decrease	46.7
2212	215	1	14	101	0.14	0.02	1.1E-08	1.3E-05	0.0044	2.8056	0.026	p53 loss increase	26.9
2212	261	1	25	100	0.25	0.08	4.1E-07	4.8E-04	0.0051	2.2885	0.025	p53 loss increase	33.1
2222	261	1	63	100	0.63	0.86	2.0E-08	2.4E-05	-0.0068	-3.0827	-0.004	Normal genotype decrease	33.1
2222	2	1	59	65	0.91	0.58	8.0E-09	9.3E-06	0.0039	1.0646	0.009	Normal genotype increase	83.5
2221	2	1	0	65	0.00	0.21	3.3E-07	3.9E-04	-0.0025	-0.6823	-0.005	p16 loss decrease	83.5
2221	11	1	15	97	0.15	0.01	2.4E-12	2.8E-09	0.0034	1.5161	0.018	p16 loss increase	42.3
1222	11	1	0	97	0.00	0.12	1.7E-05	1.9E-02	-0.0027	-1.2335	-0.005	CEP17 loss decrease	42.3
2222	15	2	77	94	0.82	0.95	4.0E-06	4.6E-03	-0.0056	-0.5040	-0.004	Normal genotype decrease	23.5
2221	17	1	0	120	0.00	0.13	8.7E-08	1.0E-04	-0.0020	-0.3571	-0.004	p16 loss decrease	67.2
2222	17	2	97	100	0.97	0.80	1.0E-06	1.2E-03	0.0025	0.4581	0.006	Normal genotype increase	67.2
2222	21	1	91	95	0.96	0.62	1.2E-14	1.4E-11	0.0139	0.6268	0.029	Normal genotype increase	24.4
3322	21	1	0	95	0.00	0.27	1.4E-13	1.6E-10	-0.0111	-0.5035	-0.022	CEP17 and Her2 gain decrease	24.4
2322	21	2	0	89	0.00	0.17	1.8E-07	2.1E-04	-0.0068	-0.3093	-0.014	CEP17 gain decrease	24.4
2222	27	1	94	101	0.93	0.67	8.0E-10	9.4E-07	0.0073	0.6574	0.016	Normal genotype increase	35.9
2221	27	1	7	101	0.07	0.23	2.6E-05	3.1E-02	-0.0045	-0.4053	0.002	p16 loss decrease	35.9
2221	33	1	0	78	0.00	0.16	1.6E-06	1.9E-03	-0.0039	-2.4761	-0.008	p16 loss decrease	41.9
2222	33	1	78	78	1.00	0.73	2.9E-11	3.4E-08	0.0065	4.1268	0.013	Normal genotype increase	41.9
2222	37	1	79	79	1.00	0.72	9.1E-12	1.1E-08	0.0034	0.6152	0.007	Normal genotype increase	81.7
2221	37	1	0	79	0.00	0.18	2.1E-07	2.5E-04	-0.0022	-0.3999	-0.004	p16 loss decrease	81.7
2222	47	2	86	101	0.85	0.99	5.9E-13	6.9E-10	-0.0023	-1.4490	-0.002	Normal genotype decrease	60.1

2222	56	1	59	61	0.97	0.76	1.6E-05	1.9E-02	0.0056	4.0259	0.012	Normal genotype increase	37.4
2222	63	1	68	79	0.86	0.98	2.1E-07	2.4E-04	-0.0016	-0.4461	-0.001	Normal genotype decrease	73.7
2222	73	1	93	100	0.93	0.69	1.2E-08	1.4E-05	0.0068	1.8466	0.016	Normal genotype increase	34.9
2222	78	2	92	100	0.92	0.99	8.2E-06	9.6E-03	-0.0117	-2.1184	-0.010	Normal genotype decrease	6.0
2222	82	1	92	102	0.90	0.99	9.2E-08	1.1E-04	-0.0029	-0.1302	-0.003	Normal genotype decrease	30.6
2221	91	1	0	100	0.00	0.11	2.5E-05	3.0E-02	-0.0032	-0.1439	-0.006	p16 loss decrease	33.3
2222	96	1	90	100	0.90	0.99	7.6E-08	8.9E-05	-0.0024	-0.1073	-0.002	Normal genotype decrease	37.9
2221	105	1	0	102	0.00	0.13	1.1E-06	1.3E-03	-0.0018	-0.6411	-0.004	p16 loss decrease	75.3
2222	112	2	94	100	0.94	0.69	2.6E-09	3.0E-06	0.0060	0.2717	0.013	Normal genotype increase	40.9
2222	117	1	99	100	0.99	0.84	1.3E-06	1.6E-03	0.0041	0.1857	0.008	Normal genotype increase	35.9
2221	130	1	0	78	0.00	0.23	2.3E-09	2.7E-06	-0.0064	-2.8993	-0.013	p16 loss decrease	36.0
2222	130	1	78	78	1.00	0.69	4.6E-13	5.4E-10	0.0085	3.8657	0.017	Normal genotype increase	36.0
2222	133	2	50	64	0.78	0.99	3.0E-15	3.5E-12	-0.0059	-0.5298	-0.006	Normal genotype decrease	35.6
2221	152	1	3	63	0.05	0.27	1.1E-05	1.3E-02	-0.0070	-0.6352	-0.003	p16 loss decrease	31.5
2222	154	2	78	86	0.91	0.99	2.7E-06	3.1E-03	-0.0022	-0.1992	-0.002	Normal genotype decrease	37.7
2222	158	2	61	68	0.90	0.99	5.7E-06	6.6E-03	-0.0029	-0.1318	-0.003	Normal genotype decrease	31.9
2221	165	1	9	87	0.10	0.01	4.7E-06	5.4E-03	0.0014	0.6242	0.010	p16 loss decrease	64.6
2221	171	1	0	109	0.00	0.15	6.7E-08	7.9E-05	-0.0039	-1.7683	-0.008	p16 loss decrease	37.3
2222	185	2	92	100	0.92	0.99	8.2E-06	9.6E-03	-0.0020	-0.8827	-0.002	Normal genotype decrease	35.9
2222	194	1	75	89	0.84	0.99	3.8E-13	4.4E-10	-0.0049	-0.4453	-0.005	Normal genotype decrease	29.9
2221	195	1	0	98	0.00	0.16	1.4E-07	1.6E-04	-0.0023	-0.2120	-0.005	p16 loss decrease	66.2
2222	200	2	97	117	0.83	0.96	1.2E-08	1.5E-05	-0.0049	-0.2227	-0.004	Normal genotype decrease	27.2
2222	213	2	95	100	0.95	0.79	1.8E-05	2.1E-02	0.0014	0.0627	0.003	Normal genotype increase	112.8
2222	217	1	75	87	0.86	0.99	4.1E-11	4.8E-08	-0.0014	-0.0630	-0.001	Normal genotype decrease	92.3
2222	218	1	90	90	1.00	0.67	1.9E-16	2.2E-13	0.0095	2.5714	0.019	Normal genotype increase	35.2
2221	218	1	0	90	0.00	0.21	1.5E-09	1.8E-06	-0.0059	-1.5918	-0.012	p16 loss decrease	35.2
2222	229	2	57	70	0.81	0.99	2.8E-13	3.3E-10	-0.0030	-0.8038	-0.003	Normal genotype decrease	59.3
2222	230	2	75	77	0.97	0.81	2.9E-05	3.4E-02	0.0060	1.0850	0.013	Normal genotype increase	27.2
2222	244	1	72	100	0.72	0.90	3.5E-07	4.1E-04	-0.0048	-7.7454	-0.003	Normal genotype decrease	37.8

2221	248	1	0	100	0.00	0.11	1.6E-05	1.9E-02	-0.0026	-0.7150	-0.005	p16 loss decrease	41.2
2222	248	1	98	100	0.98	0.66	3.0E-15	3.5E-12	0.0077	2.0925	0.016	Normal genotype increase	41.2
2222	248	2	95	101	0.94	0.74	3.0E-07	3.6E-04	0.0049	1.3226	0.011	Normal genotype increase	41.2
2221	256	1	0	120	0.00	0.10	1.3E-05	1.6E-02	-0.0027	-1.7307	-0.005	p16 loss decrease	35.2
2222	256	1	117	120	0.98	0.85	6.2E-06	7.2E-03	0.0037	2.3192	0.008	Normal genotype increase	35.2
2222	259	1	55	71	0.77	0.93	1.4E-05	1.7E-02	-0.0027	-0.1212	-0.002	Normal genotype decrease	59.2
2222	262	1	71	93	0.76	0.91	2.1E-05	2.4E-02	-0.0056	-3.0615	-0.003	Normal genotype decrease	26.0
1222	266	2	0	100	0.00	0.10	4.0E-05	4.6E-02	-0.0016	-0.0741	-0.003	CEP7 loss decrease	61.0
2222	266	2	100	100	1.00	0.86	6.5E-07	7.6E-04	0.0023	0.1038	0.005	Normal genotype increase	61.0
2222	278	2	94	105	0.90	0.99	1.1E-08	1.2E-05	-0.0029	-1.5978	-0.003	Normal genotype decrease	32.2
2222	281	2	102	104	0.98	0.76	2.6E-10	3.0E-07	0.0144	0.6510	0.030	Normal genotype increase	15.5
2221	285	1	9	116	0.08	0.25	1.8E-06	2.1E-03	-0.0026	-0.4699	0.001	p16 loss decrease	67.7
2222	285	1	100	116	0.86	0.62	1.3E-08	1.5E-05	0.0036	0.6473	0.009	Normal genotype increase	67.7
2222	286	1	53	71	0.75	0.96	2.8E-10	3.2E-07	-0.0062	-4.5102	-0.006	Normal genotype decrease	34.3
2221	288	1	0	100	0.00	0.10	3.9E-05	4.6E-02	-0.0028	-0.1274	-0.006	p16 loss decrease	35.9
2221	293	1	0	124	0.00	0.09	8.3E-06	9.6E-03	-0.0011	-0.5196	-0.002	p16 loss decrease	81.9
2222	293	2	110	119	0.92	0.99	3.6E-06	4.2E-03	-0.0008	-0.3624	-0.001	Normal genotype decrease	81.9
2222	316	2	95	100	0.95	0.77	2.0E-06	2.3E-03	0.0049	0.2227	0.011	Normal genotype increase	36.6
2222	320	1	88	88	1.00	0.83	1.0E-07	1.2E-04	0.0047	3.0025	0.009	Normal genotype increase	36.1

Supplementary Table 3: p16 loss dynamics between the first and third time points in 90 patients. P-values were obtained using binomial tests.

PatientID	Difference TP 1 to 3	p	Bonferroni
121	-0.06	2.4E-02	1.0E+00
2	-0.21	5.9E-11	4.2E-09
86	0.01	NA	NA
179	0.00	NA	NA
253	0.00	NA	NA
151	-0.13	4.5E-05	3.2E-03
58	0.01	NA	NA
53	-0.05	2.2E-02	1.0E+00
186	-0.02	3.8E-01	1.0E+00
264	0.00	1.0E+00	1.0E+00
118	-0.01	8.0E-01	1.0E+00
319	-0.05	3.2E-02	1.0E+00
290	0.01	NA	NA
190	-0.02	2.8E-01	1.0E+00
116	0.02	NA	NA
130	-0.25	2.0E-11	1.4E-09
159	-0.05	2.2E-02	1.0E+00
225	-0.11	2.0E-04	1.4E-02
152	-0.27	3.5E-14	2.5E-12
235	-0.06	3.0E-03	2.1E-01
167	0.07	NA	NA
228	-0.10	2.7E-04	1.9E-02
294	-0.03	1.8E-01	1.0E+00
197	-0.03	5.3E-01	1.0E+00
131	-0.08	9.8E-04	7.0E-02
234	-0.09	1.2E-02	8.6E-01
200	-0.02	4.9E-01	1.0E+00
237	-0.08	6.4E-04	4.6E-02
302	-0.07	3.2E-03	2.2E-01
99	0.02	NA	NA
132	-0.04	6.5E-02	1.0E+00
34	0.01	NA	NA
62	-0.02	5.6E-01	1.0E+00
133	-0.05	2.2E-02	1.0E+00
40	-0.07	1.5E-03	1.1E-01
239	-0.07	7.5E-04	5.3E-02
205	-0.07	1.7E-02	1.0E+00
15	-0.13	8.7E-06	6.2E-04
268	-0.01	8.0E-01	1.0E+00
305	-0.03	9.4E-02	1.0E+00
70	-0.08	1.6E-04	1.1E-02
91	-0.14	2.2E-06	1.5E-04
13	-0.06	1.7E-02	1.0E+00
307	-0.03	1.8E-01	1.0E+00
12	-0.18	5.0E-09	3.5E-07
248	-0.14	2.4E-07	1.7E-05
316	-0.02	5.6E-01	1.0E+00
44	0.03	NA	NA
135	-0.03	9.4E-02	1.0E+00
174	0.00	1.0E+00	1.0E+00
256	-0.11	3.2E-04	2.3E-02
182	-0.09	9.9E-05	7.1E-03
288	-0.08	4.0E-03	2.8E-01
153	-0.02	5.3E-01	1.0E+00
273	-0.04	3.6E-02	1.0E+00
120	-0.09	4.3E-04	3.1E-02
275	-0.01	8.5E-01	1.0E+00
88	0.03	NA	NA
223	0.30	7.4E-23	5.2E-21
293	-0.07	4.0E-03	2.8E-01
138	-0.06	1.3E-03	9.3E-02

216	-0.04	9.5E-02	1.0E+00
214	-0.04	6.7E-02	1.0E+00
278	-0.07	1.4E-03	9.6E-02
165	-0.01	6.5E-01	1.0E+00
155	-0.13	2.5E-06	1.8E-04
287	-0.05	3.6E-02	1.0E+00
94	-0.11	1.7E-04	1.2E-02
52	-0.02	2.8E-01	1.0E+00
16	-0.05	1.5E-02	1.0E+00
208	-0.12	6.6E-06	4.7E-04
31	-0.06	1.3E-03	9.2E-02
263	-0.06	1.2E-02	8.5E-01
98	0.04	NA	NA
128	0.01	NA	NA
104	-0.01	1.0E+00	1.0E+00
112	-0.06	6.1E-03	4.3E-01
285	-0.27	1.0E-13	7.4E-12
149	-0.08	1.4E-03	1.0E-01
66	-0.11	2.7E-05	1.9E-03
100	-0.12	3.2E-05	2.3E-03
127	0.04	NA	NA
102	-0.09	6.3E-05	4.5E-03
194	0.02	NA	NA
306	0.01	NA	NA
50	0.14	1.1E-04	7.7E-03
51	0.20	1.7E-10	1.2E-08
106	0.01	NA	NA

Supplementary Table 4: Multivariate analyses including clinical factors age and circumferential length (C length) and variables significant in univariate analyses (Table 2).

Model	Variable	Coefficient	Hazard ratio	95% Confidence interval	P-value
Clinical only	C length	0.090	1.094	0.972 - 1.231	0.1362
	Age	0.046	1.047	1.004 - 1.092	**0.0307**
+ Number of clones per cell (Set 2)	C length	0.105	1.111	0.982 - 1.257	0.0962
	Age	0.047	1.048	1.004 - 1.094	**0.0336**
	Number of clones/cell	0.192	1.211	1.068 - 1.375	**0.0030**
+ CEP7 only Shannon diversity	C length	0.055	1.057	0.936 - 1.193	0.3738
	Age	0.052	1.053	1.008 - 1.100	**0.0201**
	CEP7 diversity	0.534	1.705	1.165 - 2.495	**0.0060**
+ Number of clones per cell (Set 1)	C length	0.079	1.082	0.959 - 1.222	0.2011
	Age	0.050	1.051	1.006 - 1.100	**0.0277**
	Number of clones/cell	0.251	1.286	1.075 - 1.538	**0.0059**
+ Avg pw Distance (Set 2)	C length	0.094	1.099	0.974 - 1.239	0.1245
	Age	0.047	1.048	1.004 - 1.095	**0.0331**
	Avg pw distance	0.343	1.409	1.093 - 1.815	**0.0080**
+ Myc only Shannon diversity	C length	0.097	1.102	0.975 - 1.246	0.1203
	Age	0.046	1.047	1.002 - 1.093	**0.0400**
	Myc diversity	0.503	1.654	1.124 - 2.435	**0.0107**
+ Shannon diversity (Set 2)	C length	0.096	1.100	0.976 - 1.241	0.1192
	Age	0.048	1.049	1.004 - 1.096	**0.0321**
	Shannon diversity	0.198	1.219	1.046 - 1.421	**0.0112**
+ Aneusomy	C length	0.074	1.076	0.955 - 1.214	0.2299
	Age	0.056	1.058	1.012 - 1.106	**0.0134**
	Aneusomy	0.145	1.156	1.042 - 1.282	**0.0061**
+ Simpson diversity (Set 2)	C length	0.093	1.098	0.974 - 1.237	0.1273
	Age	0.047	1.048	1.004 - 1.095	**0.0333**
	Simpson diversity	0.443	1.558	1.057 - 2.296	**0.0251**

Supplementary Table 5: Genetic diversity-based risk stratification.

**Variable**	**Cut-off**	**Raw p**	**Corrected p**	**Sensitivity**	**Specificity**
Average pairwise distance (Set 2)	top 25%	0.0004	***0.0072***	0.55	0.77
Simpson diversity (Set 2)	top 25%	0.0005	***0.0095***	0.55	0.77
Shannon diversity (Set 2)	top 25%	0.0006	***0.0101***	0.55	0.77
Number of clones per cell (Set 2)	top 25%	0.0009	***0.0158***	0.50	0.78
Number of clones per cell (Set 1)	top 25%	0.0012	***0.0216***	0.50	0.77
CEP7-only Shannon diversity	top 25%	0.0016	***0.0288***	0.55	0.77
MYC-only Shannon diversity	top 25%	0.0025	***0.0450***	0.50	0.77
Simpson diversity (Set 2)	> median	0.0030	0.0540	0.80	0.53
Aneusomy	top 25%	0.0031	0.0558	0.50	0.77
Shannon diversity (Set 2)	> median	0.0034	0.0612	0.80	0.52
Aneusomy	> median	0.0053	0.0954	0.65	0.65
MYC-only Shannon diversity	> median	0.0093	0.1674	0.60	0.66
Average pairwise distance (Set 2)	> median	0.0190	0.3420	0.75	0.52
CEP7-only Shannon diversity	> median	0.0240	0.4320	0.70	0.56
Number of clones per cell (Set 2)	> median	0.0360	0.6480	0.70	0.52
Age	> median	0.0590	1.0000	0.60	0.55
Age	top 25%	0.0790	1.0000	0.35	0.77
Number of clones per cell (Set 1)	> median	0.1600	1.0000	0.65	0.51

Supplementary Table 6: Genetic diversity-based risk stratification using 2nd time point values with follow up data and thresholds from the 1st time point.

Variable	Cut-off (1st time point values)	Raw p	Corrected p	Sensitivity	Specificity
Number of clones per cell (Set 1)	top 25%	9.7E-07	***1.7E-05***	0.79	0.77
Number of clones per cell (Set 2)	top 25%	1.1E-06	***2.0E-05***	0.79	0.73
Shannon diversity (Set 2)	top 25%	2.8E-06	***5.0E-05***	0.79	0.76
Simpson diversity (Set 2)	top 25%	1.2E-04	***2.2E-03***	0.71	0.75
Average pairwise distance (Set 2)	top 25%	7.0E-04	***1.3E-02***	0.64	0.75
CEP7-only Shannon diversity	top 25%	1.6E-03	***2.9E-02***	0.79	0.56
CEP7-only Shannon diversity	> median	1.6E-03	***2.9E-02***	0.79	0.56
Shannon diversity (Set 2)	> median	1.8E-03	***3.2E-02***	0.93	0.46
Number of clones per cell (Set 2)	> median	1.9E-03	***3.4E-02***	0.93	0.44
Simpson diversity (Set 2)	> median	2.4E-03	***4.3E-02***	0.93	0.47
Number of clones per cell (Set 1)	> median	3.1E-03	5.6E-02	0.93	0.47
Average pairwise distance (Set 2)	> median	3.2E-03	5.8E-02	0.93	0.45
Aneusomy	top 25%	1.1E-02	2.0E-01	0.50	0.79
MYC-only Shannon diversity	top 25%	1.8E-02	3.2E-01	0.32	0.57
MYC-only Shannon diversity	> median	1.8E-02	3.2E-01	0.32	0.57
Aneusomy	> median	1.9E-02	3.4E-01	0.64	0.68
Age	top 25%	1.2E-01	1.0E+00	0.36	0.77
Age	> median	1.3E-01	1.0E+00	0.57	0.56
